# A Survey of Marine Natural Compounds and Their Derivatives with Anti-Cancer Activity Reported in 2012

**DOI:** 10.3390/molecules20047097

**Published:** 2015-04-20

**Authors:** Wamtinga Richard Sawadogo, Rainatou Boly, Claudia Cerella, Marie Hélène Teiten, Mario Dicato, Marc Diederich

**Affiliations:** 1 Laboratoire de Biologie Moléculaire et Cellulaire de Cancer, Hôpital Kirchberg, 9, rue Edward Steichen, L-2540 Luxembourg, Luxembourg; E-Mails: richard.sawadogo@lbmcc.lu (W.R.S.); claudia.cerella@lbmcc.lu (C.C.); marie_helene.teiten@lbmcc.lu (M.H.T.); mdicato@gmail.com (M.D.); 2 Institut de Recherche en Sciences de la Santé (IRSS), 03 BP 7192 Ouagadougou 03, Burkina Faso; E-Mail: rainatoub@gmail.com; 3 College of Pharmacy, Seoul National University, Seoul 151-742, Korea

**Keywords:** anticancer molecules, marine origin, synthetic derivatives, cancer

## Abstract

Although considerable effort and progress has been made in the search for new anticancer drugs and treatments in the last several decades, cancer remains a major public health problem and one of the major causes of death worldwide. Many sources, including plants, animals, and minerals, are of interest in cancer research because of the possibility of identifying novel molecular therapeutics. Moreover, structure-activity-relationship (SAR) investigations have become a common way to develop naturally derived or semi-synthetic molecular analogues with improved efficacy and decreased toxicity. In 2012, approximately 138 molecules from marine sources, including isolated compounds and their associated analogues, were shown to be promising anticancer drugs. Among these, 62% are novel compounds. In this report, we review the marine compounds identified in 2012 that may serve as novel anticancer drugs.

## 1. Introduction

Cancer is a growing public health problem, particularly in developed countries, despite advances in biomedical research and technology [1,2]. According to the World Health Organization (WHO), the incidence of this disease is about 6 million cases per year. In 2012, the annual cancer cases were 14 million and this number will increase to reach 22 million within the next two decades. Moreover, cancer is a leading cause of death worldwide, accounting for 8.2 million deaths in 2012 [3]. Accordingly, there is an urgent need to identify new compounds with anticancer activity. Anticancer molecules have been isolated from animals, plants, and minerals, as well as chemically synthesized. However, owing to drug resistance, high toxicity, and unwanted side effects observed with synthetic drugs, many researchers have focused their efforts toward natural products, especially from marine environments, to identify novel anticancer compounds. Today, it is estimated that more than 60% of commercially available anticancer drugs are of natural origin [4].

The oceans cover approximately 70% of the Earth’s surface. Marine environments are a unique reservoir for bioactive natural products, with structural features not generally found in terrestrial plant metabolites. These compounds are produced by marine organisms to protect against predators, communicate and reproduce. In the last decade, more than 3000 new compounds have been discovered in marine environments, indicating that marine environments may offer a variety of novel therapeutic molecules. Among these, a number of novel marine compounds are isolated and tested for anticancer activity [5]. Thus, several natural compounds originating or derived from marine life are now undergoing clinical trials. However, very few anti-cancer drugs currently on the market are derived from marine life.

In 2011 and 2013, we published two reviews of marine compounds and their derivatives identified in 2010 and 2011, respectively, that were reported to be potential anticancer drug candidates [6,7]. In this report, we review 138 pharmacologically active compounds of marine origin that were described in 2012 as potential leads in anticancer therapy.

## 2. Marine Anticancer Molecules Reported in 2012

### 2.1. Alkaloids

#### 2.1.1. Agelasine B

**Figure 1:**
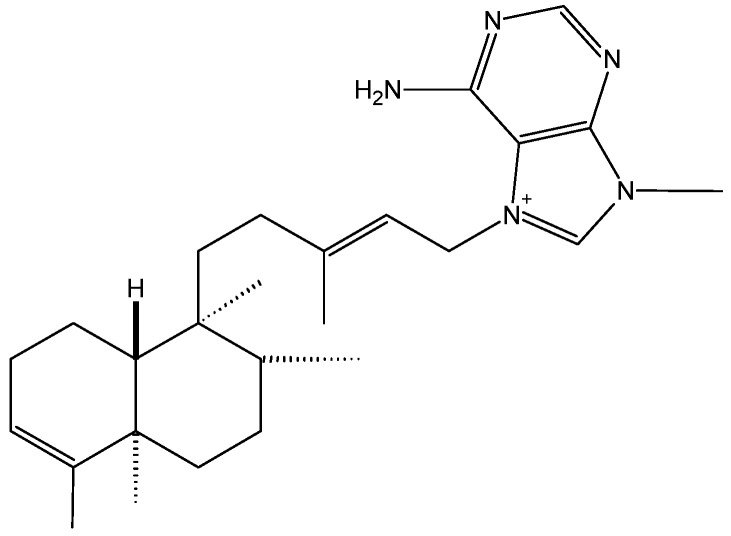
Chemical structure of agelasine B (1).

The agelasines are toxins isolated from marine sponges, first reported by Nakamura *et al.* [8]. These compounds are mono or bi-cyclic diterpenoids linked to a 9-methyladeninium chromophore. Agelasine analogs 2F and 2G were reported in 2011 as highly cytotoxic compounds against a panel of cancer cells, but their molecular mechanisms of action were not elucidated [6,9]. In 2012, Pimentel and colleagues reported the cytotoxicity of agelasine B (**1**, Figure 1) and its probable mechanism of action. In this study, agelasine B was purified from the marine sponge *Agelas clathrodes* [10]. This compound exhibited higher toxicity in cancer cells (IC_50_ = 3.22, 2.99, and 6.86 μMin MCF-7, SKBr3, and PC-3 cells, respectively) than in normal cells (fibroblasts, IC_50_ = 32.91 μM). Moreover in these cancer cells, agelasine B increased the intra-cellular concentration of Ca^2+^ and induced fast Ca^2+^ release via the endoplasmic reticulum (ER). This study demonstrated that agelasine B inhibits sarcoplasmic-ER Ca^2+^-ATPase (SERCA) activity. Intracellular Ca^2+^ accumulation, especially in the mitochondria, is highly associated with apoptosis [11,12,13,14]. In addition, this marine sponge toxin induces DNA fragmentation and significantly increases caspase-8 activity in MCF-7 cells. As a result, agelasine B is of interest for the treatment of breast cancer, since it is 10-fold less toxi-c in normal breast cells.

#### 2.1.2. Granulatimide and Isogranulatimide Analogs

**Figure 2:**
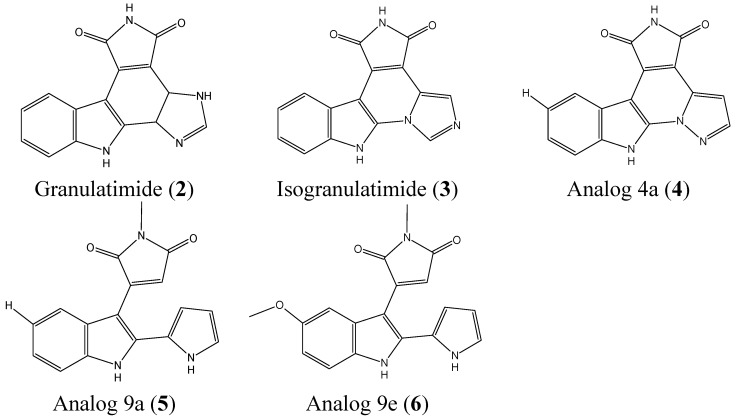
Chemical structure of granulatimide, isogranulatimide, and their analogs.

Granulatimide (**2**, Figure 2) and isogranulatimide (**3**, Figure 2) are marine alkaloids isolated from the ascidian *Didemnum granulatum* that can serve as cell cycle G2/M checkpoint inhibitors [15,16,17]. Deslandes *et al.* reported the synthesis and biological evaluation of 23 analogs of granulatimide and isogranulatimide with a particular focus on three analogs, 4a (**4**, Figure 2), 9a (**5**, Figure 2), and 9e (**6**, Figure 2), which were the most potent compounds [18]. These analogs inhibit the growth of a panel of cancer cells, including A549, U373, LoVo, MCF-7, HS683, PC-3, OE21, and B16F10 cells. Analog 4a is the most active compound with IC_50_ values ranging from 0.2 to 8 μM against A549, U373, LoVo, HS683, OE21 cells. Analog 9a was also a potent inhibitor, with IC_50_ values from 2 to 9 μM in A549, U373, LoVo, PC-3, and B16F10 cells, whereas 9e had an IC_50_ ranging from 6 to 10 μM in A549, U373, MCF-7, and B16F10 cells. The three analogs are cytostatic, and not cytotoxic. Only 4a showed significant G2 checkpoint abrogation activity, which was comparable to that of granulatimide [18].

#### 2.1.3. Bis(indolyl)hydrazide-hydrazone Analogs

**Figure 3:**
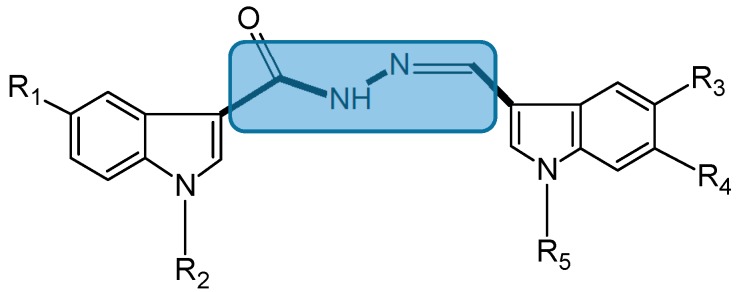
Chemical structure of bis(indolyl)hydrazide-hydrazone analogs.

Bis(indole) alkaloids were discovered in marine invertebrates, including sponges and tunicates [19,20]. Kumar *et al.* synthesized a series of 14 bis(indolyl)hydrazide-hydrazones, which were evaluated for cytotoxicity in six cancer cell lines, including prostate (PC-3, DU145, and LnCaP), breast (MCF and MDA-MB-231), and pancreatic (PaCa2) cancer [21]. Among these compounds, analog 5b (**7**, Figure 3) had the highest cytotoxicity against DU145, LnCaP, MCF, MDA-MB-231, and PaCa2 cells, with IC_50_ values ranging from 1 to 8.7 μM. This was followed by analog 5k (**8**, Figure 3), which was selective against MCF7 cells (IC_50_ = 3.1 μM), 5e (**9**, Figure 3), which was selective against MDA-MB-231 cells (IC_50_ = 5 μM), and 5d (**10**, Figure 3) and 5f (**11**, Figure 3), which were cytotoxic against MCF-7 and MDA-MB-231 cells, with IC_50_ values ranging from 5.1 to 8.3 μM. A structure activity relationship (SAR) study revealed that the substituents like N-(4-chlorobenzyl) as well as bromo and fluoro on the indole ring are required for the cytotoxicity activity. These compounds need to be tested on healthy cells to verify that their cytotoxicity is selective.

5b (**7**): R_1_ = R_2_ = R_4_ = H, R_3_ = Br and R_5_ = 4-ClC_6_H_4_CH_2_; 5d (**10**): R_1_ = R_2_ = R_3_ = H, R_4_ = F and R_5_ = 4-ClC_6_H_4_CH_2_; 5f (**11**): R_1_ = Br, R_2_ = R_3_ = H, R_4_ = F and R_5_ = 4-ClC_6_H_4_CH_2_; 5e (**9**): R_2_ = R_4_ = H, R_1_ = Br, R_3_ = OCH_3_ and R_5_ = 4-ClC_6_H_4_CH_2_; 5k (**8**): R_2_ = R_4_ = H, R_1_ = Br, R_3_ = OCH_3_ and R_5_ = 4-CH_3_C_6_H_4_CH_2_.

#### 2.1.4. Hyrtioreticulins A and B

**Figure 4:**
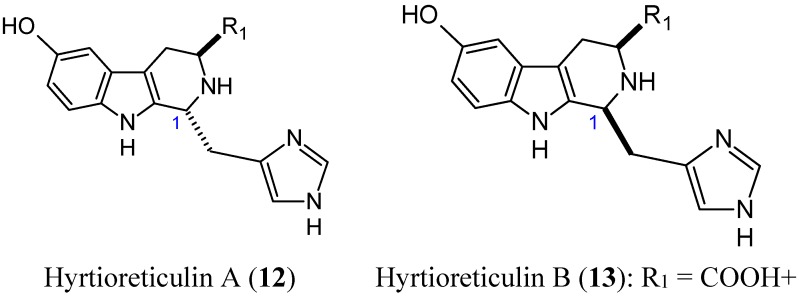
Chemical structures of hyrtiorecticulins analogs.

Hyrtioreticulins A (**12**, Figure 4) and B (**13**, Figure 4) are indole alkaloids that were isolated from the marine sponge *Hyrtios reticulatus* by Yamanokuchi and colleagues. These alkaloids significantly inhibit E1-ubiquitin intermediate formation, with IC_50_ values of 0.75 and 11 μg/mL [22]. Their chemical structures are almost the same, differing in their stereochemistry at C-1, where hyrtioreticulin A is *trans*-configured and hyrtioreticulin B is *cis.* This difference in structure suggests that the *trans* configuration enhances inhibitory activity against E1, the ubiquitin-activating enzyme, which is one of three enzymes (E1, E2, and E3) that are required for ubiquitination in the ubiquitin-proteasome pathway, which is implicated in numerous cellular events, including cell cycle control, transcription, and development [23,24]. Thus, deregulation of this pathway can lead to various diseases, including cancer. As a result, the ubiquitin pathway has emerged as an important target for anticancer drugs. In that context, hyrtioreticulin A and B are of great interest for the development of new anticancer therapeutics.

#### 2.1.5. Lamellarin D Analogs

**Figure 5:**
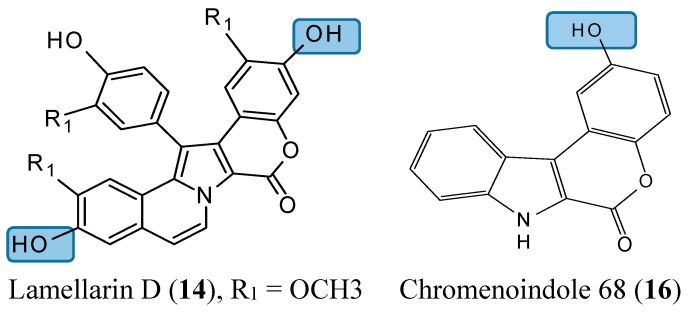

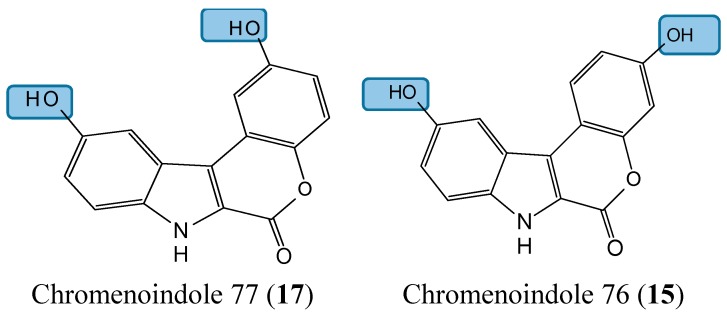
Chemical structures of chromenoindole analogs.

Lamellarin D (**14**, Figure 5) is a polyaromatic pyrrole alkaloid isolated from several marine organisms, such as mollusks, ascidians, and sponges. This compound is well known as a potent anticancer agent through its cytotoxicity, and inhibition of kinases and topoisomerase I [25]. Neagoie *et al.* synthetized 20 new compounds using the natural scaffold of lamellarin D in order to find specific inhibitors of topoisomerase I and various kinases [26]. Among them, only one compound, chromenoindole 76 (**15**, Figure 5), was shown to strongly inhibit topoisomerase I [26]. Topoisomerase I plays a key role in DNA replication by initiating the cleavage of one strand of the DNA double helix. Partial or total inhibition of this process leads to DNA strand breaks, cell cycle arrest, and apoptosis [27]. Thus, given that C-3, C-10 bis-hydroxylated chromenoindole 76 is a strong inhibitor of topoisomerase I with low toxicity (IC_50_= 38.5 μM against CEM cells), it should be an interesting scaffold for anticancer drug development. SAR studies revealed that the hydroxyl groups in position C-3 and C-10 are required for topoisomerase inhibition. Removal of these hydroxyl groups ablates the inhibitory effect. If the hydroxyl group is positioned at C-2 or at C-2 and C-10, the corresponding compounds (C-2 hydroxylated chromenoindole 68 [**16**, Figure 5] and C-2, C-10 bis-hydroxylated chromenoindole 77 [**17**, Figure 5]) selectively inhibit DYRK1A, with IC_50_ values of 74 nM and 67 nM, respectively. DYRK1A is a dual-specificity tyrosine phosphorylation-regulated kinase that has been implicated in proliferation, neurogenesis, neuronal differentiation, cell death, and synaptic plasticity [26,28]. In addition, Pozo *et al.*, demonstrated that DYRK1A inhibition promotes the degradation of epithelial growth factor receptor (EGFR) and impairs tumor growth [28]. Thus, chromenoindoles 68 and 77 are selective inhibitors of DYRK1A that may be used as lead compounds to develop drugs for oncological diseases involving this kinase.

### 2.2. Amine Derivatives

#### 5-(2,4-Dimethylbenzyl) pyrrolidin-2-one (DMBPO)

**Figure 6:**
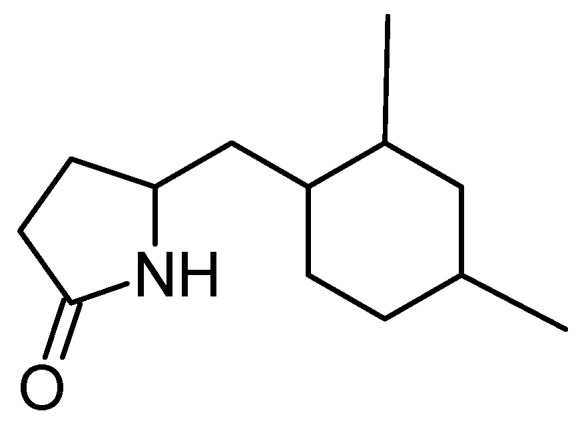
Chemical structure of 5-(2,4-dimethylbenzyl) pyrrolidin-2-one (DMBPO, **18**).

Saurav and Kannabiran isolated DMBPO (**18**, Figure 6) from marine *Streptomyces* VITSVK5 spp. [29]. DMBPO is an amine-derived compound that exhibited cytotoxic activity towards HEP2 and HepG2 cells. The inhibition was dose- and time-dependent, with the IC_50_ values of 8.3 μg/mL and 2.8 μg/mL, respectively for HEP2 and HepG2 cells.

### 2.3. Macrolides

#### 2.3.1. Biselyngbyasides

**Figure 7:**
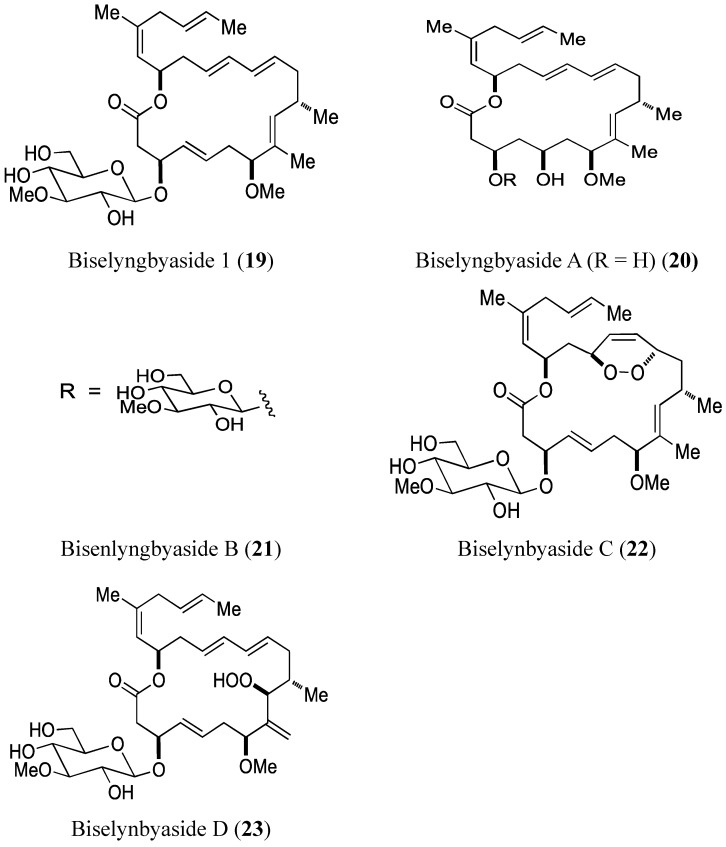
Biselyngbyasides 1, A, B, C, D.

Morita *et al.* [30] reported the isolation and structural determination of three novel analogs of biselyngbyaside 1 (**19**, Figure 7) and biselyngbyolide A (**20**, Figure 7). Biselyngbyasides B (**21**), C (**22**), and D (**23**, Figure 7) were obtained as colorless oils from the marine cyanobacterium *Lyngbya* sp. Among these novel analogs, only biselyngbyaside B (**21**) exhibited growth-inhibitory and apoptosis-inducing activity against both HeLa S_3_ cells and HL60 cells, with the best results seen in HL60 cells (IC_50_ = 0.040 and 0.82 µM, respectively). Apoptosis in HeLa S_3_ cells could be explained by the ability of biselyngbyasides (**19**–**23**) to increase intracellular Ca^2+^ levels [30].

#### 2.3.2. Bryostatin-1

**Figure 8:**
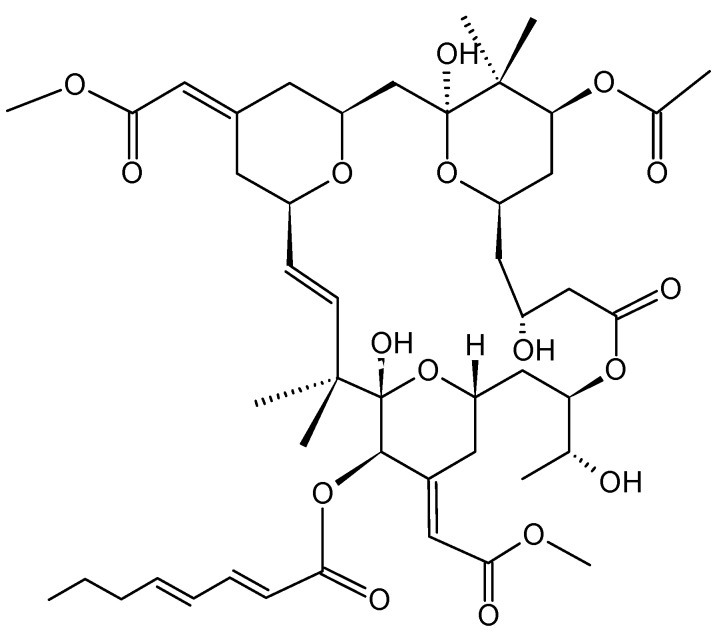
Bryostatin-1 (**24**).

Bryostatins (**24**, Figure 8) are a family group of macrolide lactones that have been isolated from the extracts of the invertebrate marine bryozoan *Bugula neritina*. According to the National Cancer Institute, bryostatin-1 has antitumor activity against various cancer cells lines, including lung, breast, ovarian, melanoma, sarcoma, lymphoma, and leukemia cell lines [31]. A phase II clinical trial combining bryostatin-1 and cisplatin (cDDP) for the treatment of patients with recurrent ovarian cancer showed a moderate response rate and severe myalgias that prevented tolerance and excluded this combination from further investigation at this dose and schedule [31]. Proposed mechanism of bryostatin-1 activity include the modulation of protein kinase C (PKC) activity, enhancement of drug-induced apoptosis, and sensitization of tumor cells to cisplatin. PKC enzymes represent a family of at least twelve isoforms that are involved in regulating the function of other proteins through the serine/threonine phosphorylation. Serine/threonine kinases are involved in various cellular events, including cell growth, cell cycle progression, differentiation, drug efflux, apoptosis, and tumor angiogenesis. The inhibition of PKC isoforms α and η by bryostatin-1 suggests that PKC isotypes can be useful targets for anti-neoplastic therapy, and that the high sensitization to cDDP suggests that bryostatin-1 can be used as a cisplatin chemomodulator to increase therapeutic potential [31].

#### 2.3.3. Eribulin

**Figure 9:**
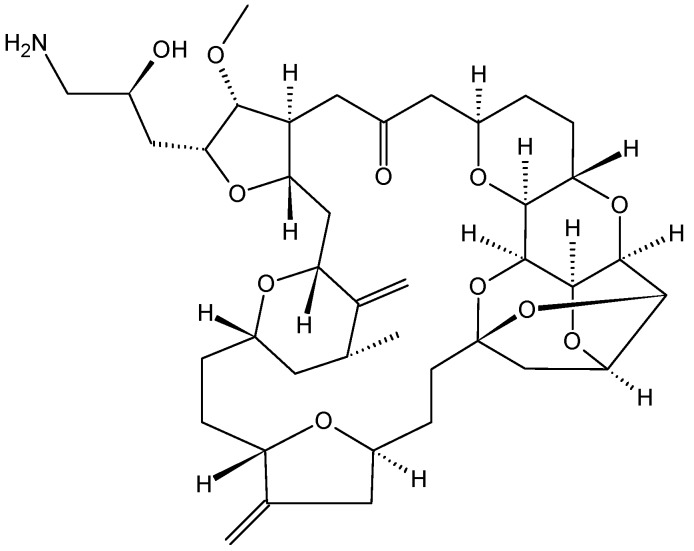
Eribulin (**25**).

Eribulin mesylate (E7389) (**25**, Figure 9) is a structurally a simplified synthetic analog of the marine natural product halichondrin B, which was isolated from the marine sponge *Halichondria okadai* and is a non-taxane microtubule dynamics inhibitor [32]. Preclinical studies have shown that eribulin elicits potent anti-proliferative effects against a broad range of human cancer cell lines, with IC_50_ values in the nanomolar range. Further, eribulin displayed significant antitumor activity in well-established human tumor xenograft models derived from breast, colon, melanoma, ovarian, and pancreatic cancer [32,33,34]. Eribulin inhibits microtubule dynamics via a novel mechanism of action that differs from other known classes of tubulin-targeted agents, such as taxanes, epothilones, and vinca alkaloid. While the later affects both growth and shortening of microtubules, eribulin binds to the microtubules and suppresses microtubule polymerization without affecting shortening, thereby sequestering tubulin into non-functional aggregates [35]. Thus, eribulin prevents the formation of mitotic spindles, leading to G2/M cell-cycle arrest and apoptosis due to prolonged mitotic blockage [34]. *In vitro* studies demonstrated that eribulin retains activity in cell lines overexpressing P-gp and that are taxane-resistant due to β-tubulin mutations [32,36]. Reports from phase I studies demonstrated that eribulin has a manageable safety profile when administrated in a 21-day cycle, and that neutropenia was the main dose-limiting toxicity, with an MTD of 1.4 mg/m^2^ [35]. Eribulin demonstrated antitumor activity in several phase II studies with good efficacy and safety profiles [33,35,36]. As a result, eribulin has been approved for the treatment of patients with locally advanced or metastatic breast cancer previously treated with at least two chemotherapeutic regimens for advanced disease [35,36].

#### 2.3.4. Halichoblelides

**Figure 10:**
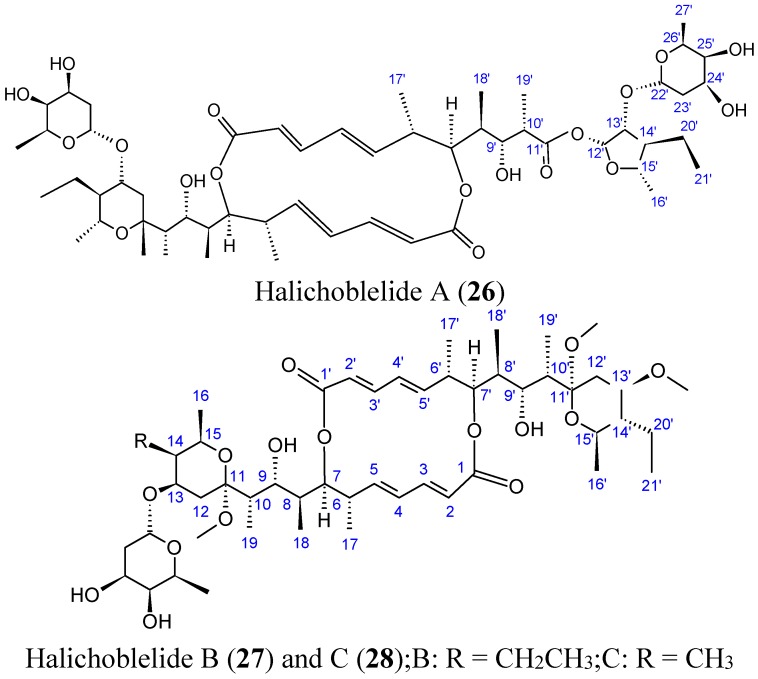
Halichoblelides A, B, C.

Halichoblelides are novel macrolides that were isolated from a strain of *Streptomyces* originally separated from the marine fish *Halichoeres bleekeri*. Their absolute stereostructures were determined by spectroscopic analyses and chemical transformations [37,38]. In 2002, Yamada and colleagues reported the isolation of halichoblelide A (**26**, Figure 10), from *Streptomyces hygroscopicus* OUPS-N92, and in 2012, two new 16-membered ring macrolides called halichoblelides B (**27**, Figure 10) and C (**28**, Figure 10) were obtained from this strain [37]. Halichoblelides A, B, and C exhibited significant cytotoxic activity against the murine P388 lymphocytic leukemia cell line, and demonstrated noticeable cytotoxicity in a panel of 39 human cancer cell lines, including breast, central nervous system, colon, lung, melanoma, and ovary cancer cell lines.

**Figure 11:**
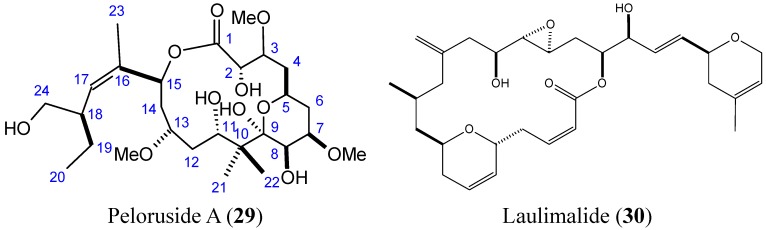
Peloruside A and Laulimalide.

The mean value of the log of GI_50_ (growth inhibition) for halichoblelide A, B, and C were −5.75, −5.72, and −5.93, respectively. Halichoblelide C (**28**, Figure 11) demonstrated selective cytotoxic activity, and the COMPARE analysis of differential cytotoxicity of this compound suggested a different mode of action than other previously developed anticancer drugs [37].

#### 2.3.5. Peloruside A and Laulimalide

Peloruside A (PLA) and laulimalide (LAU) (**29**–**30**, Figure 11) are macrolides that are structurally distinct from taxanes, which were isolated from the marine sponges *Mycale hentscheli* and *Cacospongia mycofijiense*, respectively [39,40]. PLA and LAU induced tubulin polymerization and stabilization with a paclitaxel-like mode of action, and both compounds exhibited significant anti-proliferative activity against many human cancer cell lines in the low nanomolar range [40]. PLA and LAU arrest cells in the G2-M phase of the cell cycle and induce apoptosis [39,41]. The SARs and mechanistic studies showed that the 6-membered pyranose ring of PLA and the C24 hydroxyl group were essential for biological activity [39,42]. Although their mechanism of action is similar to that of paclitaxel, PLA and LAU differ from it in terms of their binding site on β-tubulin. Moreover, PLA and LAU showed low susceptibility to the P-gp drug efflux pump compared to paclitaxel [39,42,43]. In 2012, Kanakkanthara *et al.* [43] identified a novel mechanism of resistance to PLA and LAU that involves the down-regulation of vimentin, which has several cellular functions, including regulation of cell signaling, cell division, cell survival, apoptosis, migration, and intermediate filament structure and dynamics. In fact, down-regulation of vimentin in human ovarian carcinoma cells may lead to alterations in some cellular signaling processes that regulate cell survival or apoptosis, thereby conferring resistance to PLA and LAU. Hamel *et al.* [44] demonstrated that PLA and LAU act synergistically with paclitaxel and other taxol drugs that affect tubulin assembly; however, they were unable to synergize with each other. The synergy they display highlights that these compounds as promising leads for drug development.

#### 2.3.6. Mycoepoxydiene

**Figure 12:**
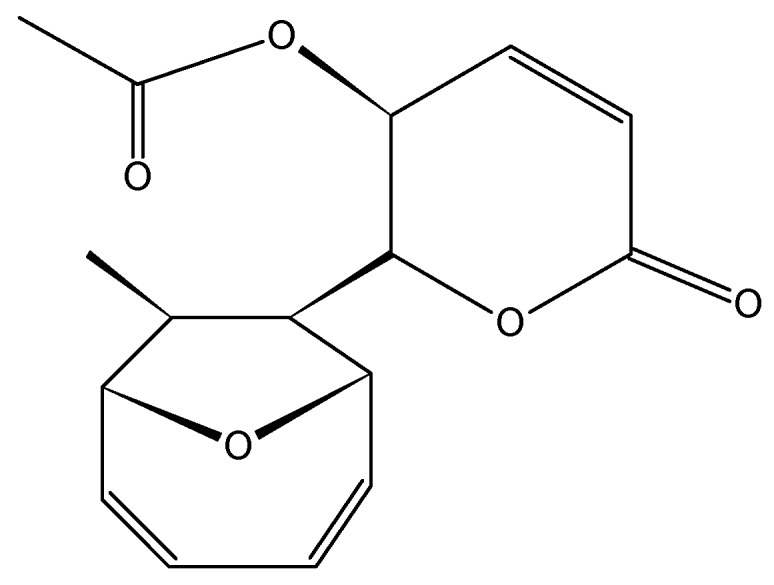
Mycoepoxydiene (MED, **31**).

Mycoepoxydiene (MED, **31**, Figure 12) is a polyketide containing an oxygen bridged cyclooctadiene core and an α,β-unsaturated δ-lactone moiety that was first isolated from the fermentation broth of OS-F66617, a fungal strain obtained from the deadwood of forests in Brazil [45]. Lin *et al.* [45] isolated MED from the marine fungus *Diaporthe* sp., which grows on the submerged rotten leaves of *Kandelia candel* in the mangrove forest in Fujian Province, China. MED elicits cytotoxicity against the human oral epidermoid carcinoma KB, with an IC_50_ less than 6.25 µg/mL, and exhibits antimicrobial and anti-inflammatory properties through the inhibition of NF-κB and MAPK pathway activation [45,46]. Wang *et al.* [47] showed that mycoepoxydiene inhibited the growth of the MCF-7 cells with an IC_50_ of 14 mM, and induced apoptosis and DNA damage through the generation of reactive oxygen species (ROS). The mechanism of action involves the activation of the tumor suppressor p53 and the inhibition of NF-κB. Both p53 and NF-κB share important roles in human cancers, especially cancers resulting from the chronic inflammation, such as colorectal cancer and renal cell carcinoma, wherein the inactivation of the p53 and NF-κB hyperactivation is a common occurrence [47].

#### 2.3.7. Salarins

**Figure 13:**
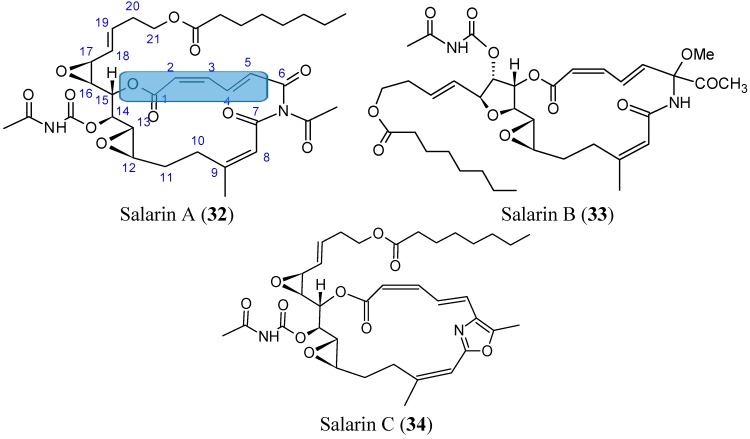
Salarins A, B, C.

Kashman *et al.* [48] isolated the salarins from the extracts of *Fascaplysinopsis* sp., a marine sponge collected from Salary Bay in Madagascar. These sponges contained four groups of nitrogenous macrolides, salarins (A–J), tulearins (A–C), taumycins (A and B), and tausalarins (C), which consist of a combination of taumycin and salarin. The salarins contain seven functional groups, which complicate the chemistry of these compounds. The cell viability, assessed by 3-(4,5-dimethylthiazol-2-yl)-2,5-diphenyltetrasolium bromide (MTT) assay, demonstrated that salarin C (**34**, Figure 13) was the most potent inhibitor of cell proliferation, since no viable cells could be detected in the culture after 3 days of treatment. Salarin B (**33**, Figure 13) inhibited proliferation approximately 50% in the chronic myeloid leukemia cell line K562, whereas salarin A (**32**, Figure 13) showed an inhibition less than 20% [49]. Salarin C induced cell cycle arrest in the G2-M phase, and thus induced apoptosis in a dose- and time-dependent manner. Furthermore, salarin C induced the cleavage of poly-ADP-ribose polymerase (PARP), caspase 3, and caspase 9, whereas the other salarins reduced cell viability, but did not cleave caspase 3. Although the mechanism by which salarin C induces apoptotic cell death has not yet been elucidated, Ben-Califa *et al.* [49] demonstrated that the effect of salarin C in both HeLa and K562 cells was rapid and irreversible, and after 4 h of treatment, the release of cytochrome C from the mitochondria was detected, indicating mitochondrial damage. The most potent activity of salarin C was related to a subtle and specific arrangement of its various functional groups. These functional moieties include a 2,4-doubly conjugated oxazole ring which was the most sensitive, a 2*Z*,4*E*-2,3,4,5-unsaturated lactone, a vinyl epoxide and another non-conjugated epoxide, as well as an acetyl carbamate [49].

#### 2.3.8. Spirastrellolides

**Figure 14:**
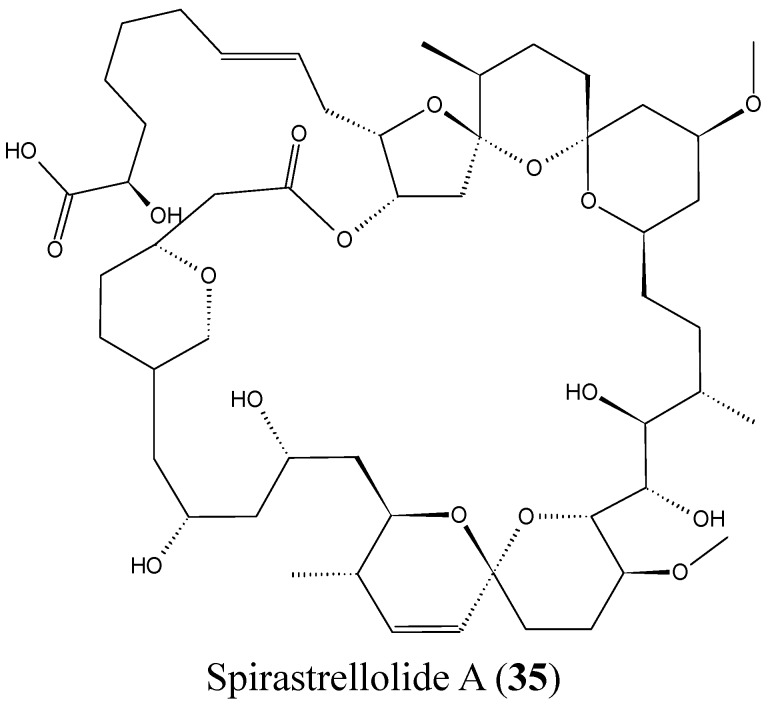

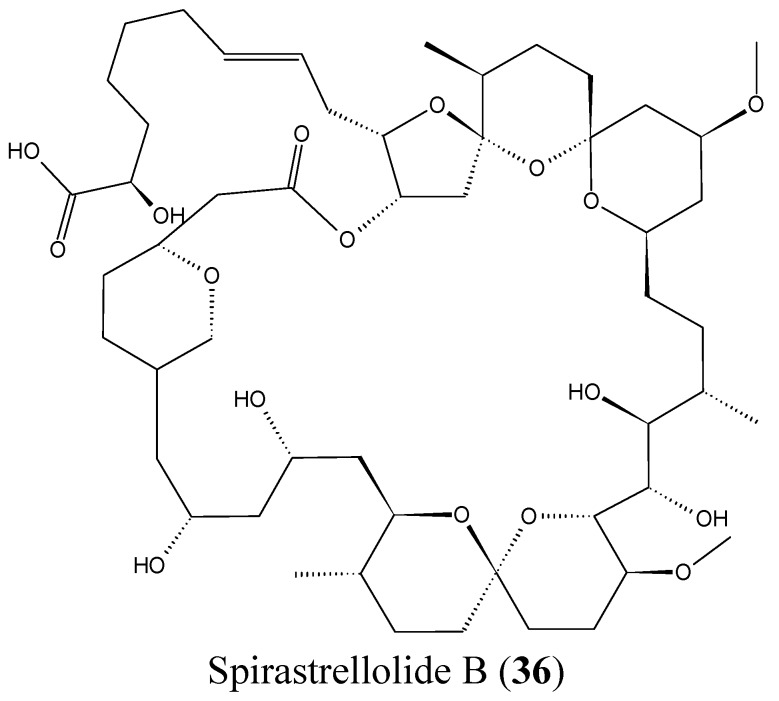
Spirastrellolides A and B.

Spirastrellolides are macrolides that were isolated by Anderson and colleagues from the Caribbean marine sponge *Spirastrella coccinea*, starting with spirastrellolide A in 2003, followed by B–G in 2007 [50]. Spirastrellolide A exhibited potent antimitotic activity at the low nanomolar range and is a strong and selective inhibitor of protein phosphatase 2A (PP2A), with a similar mode of action to other Ser/Thr phosphatase inhibitors such as fostriecin [50]. In 2012, Suzuki *et al.* reported the isolation of spirastrellolides A (**35**, Figure 14) and B (**36**, Figure 14) as free acids from a marine sponge *Epipolasis* sp. collected in the East China Sea. Spirastrellolides A and B exhibited potent cytotoxicity against HeLa cells with IC_50_ values of 20 and 40 nM [51].

### 2.4. Peptides/Polypeptides

#### 2.4.1. Cyclic Depsipeptides

**Figure 15:**
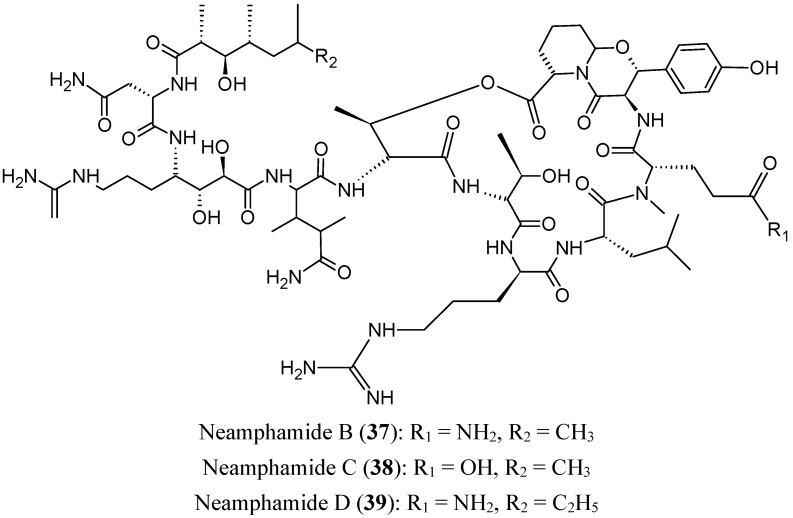
Neamphamides B, C and D.

Neamphamides B, C and D (**37**–**39**, Figure 15) are three new cyclodepsipeptides that were isolated from the Australian sponge *Neamphius huxleyi*. These three compounds demonstrated potent cytotoxic activities against a panel of human cancer and non-cancer cell lines (A549, HeLa, LNCaP, PC3, and NFF), with IC_50_ values ranging from 88 to 370 nM. Neamphamide D (**39**) should be used with caution, as it causes A549 cell proliferation at sub-cytotoxic doses [52].

**Figure 16:**
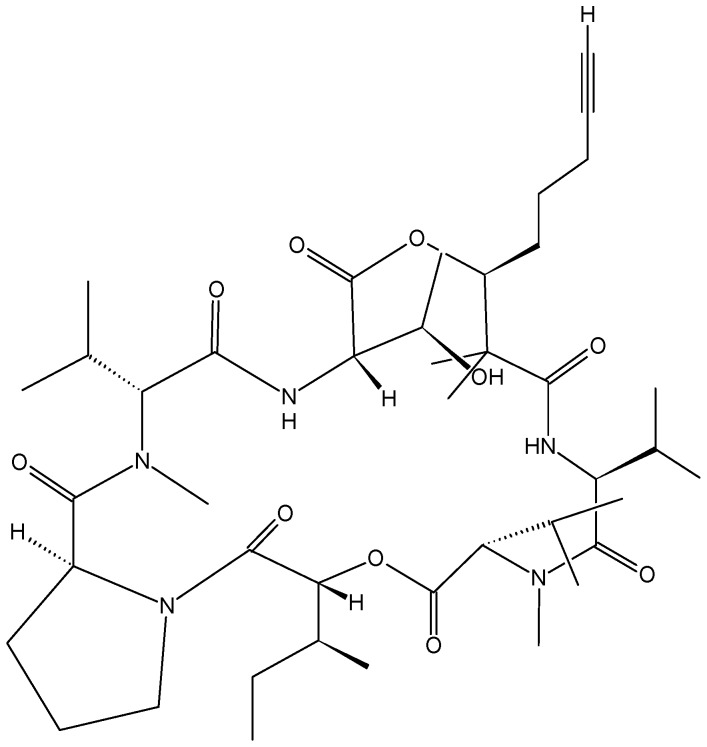
Viequeamide A.

Viequeamides, a family of 2,2-dimethyl-3-hydroxy-7-octynoic acid (Dhoya)-containing cyclic depsipeptides, were isolated from a “button” *cyanobacterium* (*Rivularia* sp.) collected from the Puerto Rican island of Vieques. These compounds are structurally related to kulolide, a metabolite originally isolated from apredatory opisthobranch mollusk. Although the viequeamides have very similar structures to each other, they showed variable cytotoxicity towards H460 human lung cancer cells. With an IC_50_ value of 60 ± 10 nM, viequeamide A (40, Figure 16) was found to be highly toxic compared to other viequeamides, which showed no toxicity [53].

**Figure 17:**
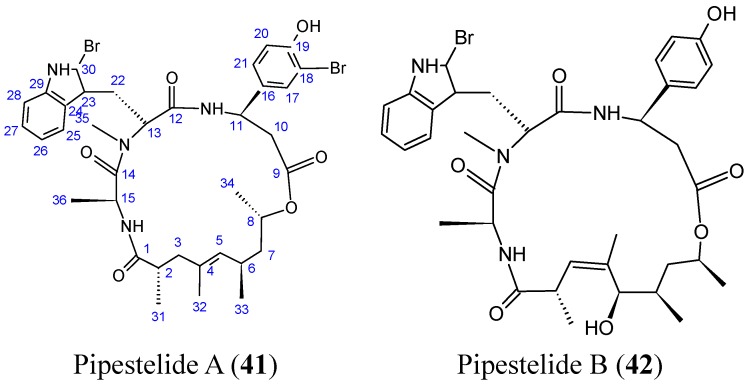

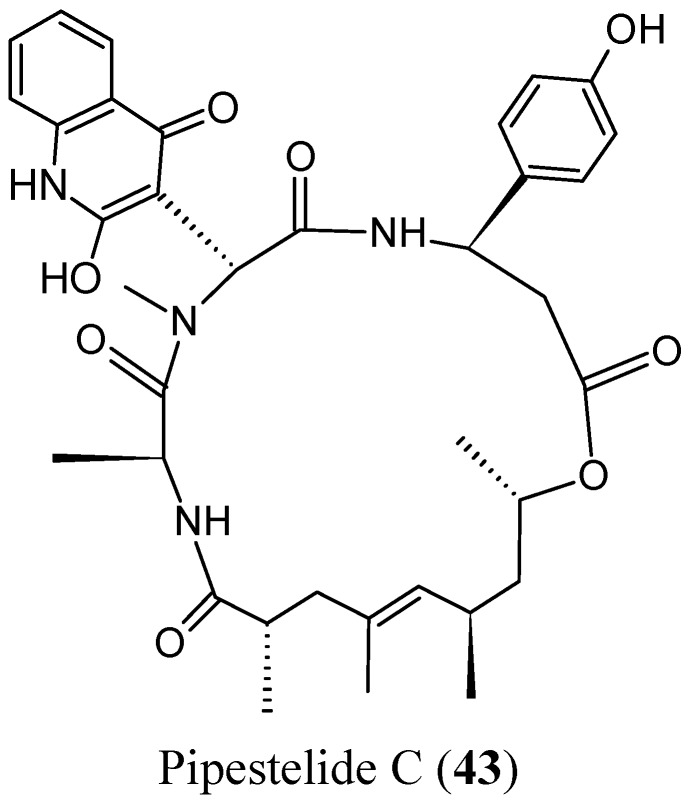
Pipestelides A, B, C.

Sorres and colleagues reported the isolation and structural elucidation of three new, unusual derivatives of jaspamide, a mixed polyketide-peptide compound often referred to as a PKS-NRPS hybrid [54]. Pipestelides A−C (**41**–**43**, Figure 17) with uncommon moieties were isolated from the Pacific marine sponge *Pipestela candelabra*. These cyclodepsipeptides exhibited significant, but variable, cytotoxicity against KB cells within the micromolar range. Pipestelide A was more active, with an IC_50_ value of 0.1 μM, whereas pipestelide B showed modest activity [54].

**Figure 18:**
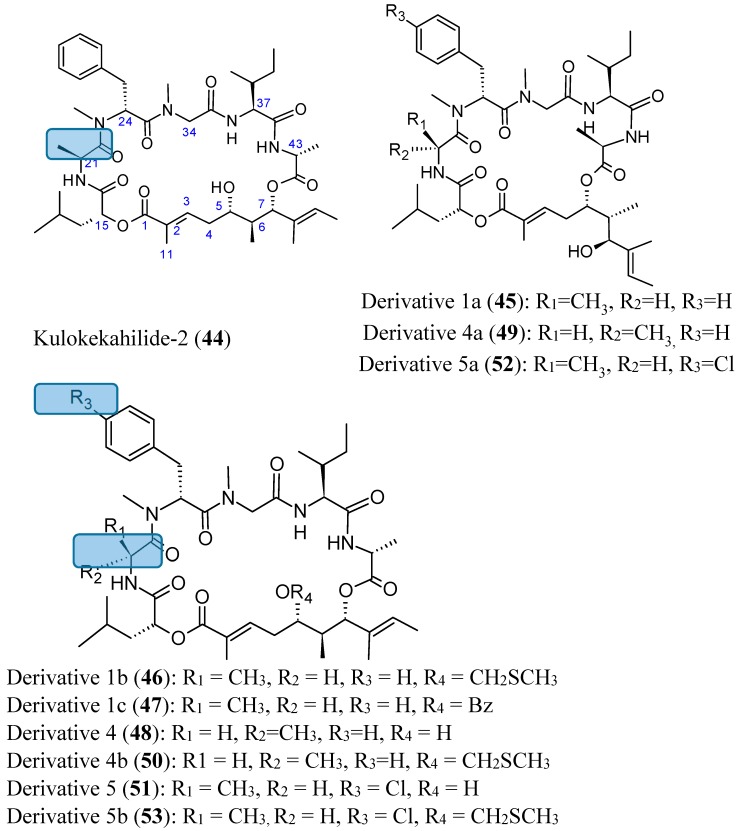
Kulokekahilide-2 and its derivatives.

Kulokekahilide-2 (**44**, Figure 18) is a 26-membered cyclodepsipeptide isolated from the Hawaiian marine mollusk *Philinopsis speciosa* that showed significant cytotoxicity in mammalian tumor cells. Synthetic kulokekahilide-2 (**44**, Figure 18) significantly inhibited 549, K562 and MCF7 cells with respective IC_50_ values of 0.0021, 0.0031, and 0.22 nM. Synthetic kulokekahilide-2 (**44**) and a 24-membered ring derivative 1a (**45**, Figure 18) demonstrated the same degree of cytotoxicity against A549, K562, and MCF7 cells, while some of its derivatives, such as derivative 4 (**48**, Figure 18), which contains 21-d-Ala instead of the 21-l-Ala in kulokekahilide-2 and the corresponding 24-membered ring derivative 4a (**49**, Figure 18) were less active. Moreover, when derivatives containing the CH_2_SCH_3_ group on 1b (**46**, Figure 18) and 4b (**50**, Figure 18) were compared, it appears that the cytotoxicity of 4b (d-Ala at the 21-position) was much lower than 1b (l-Ala at the 21-position). Derivatives 1b (**46**, Figure 18) and 1c (**47**, Figure 18), whose 5-hydroxyl group was protected, tend to have less cytotoxic activity compared to kulokekahilide-2 in K562 and MCF7 cells, whereas these derivatives were more cytotoxic than kulokekahilide-2 in A549 cells. Thus, there is a possibility that the introduction of a protecting group can cause conformational changes by steric interference in each cyclodepsipeptide (derivatives 1b and 1c), and consequently contributes to the potent cytotoxic activity towards A549 cells. The halogenated kulokekahilide-2 derivatives 5 (**51**, Figure 18) and 5a (**52**, Figure 18) which have 24-d-p-Cl-MePhe instead of 24-d-MePhe exhibited higher cytotoxicity in all three cell lines. In particular, the halogenated derivative 5 (**51**, Figure 18) was 100-fold more potent than kulokekahilide-2. Protection of the 5-hydroxyl group in the halogenated derivative 5b (**53**, Figure 18) leads to reduced cytotoxic activity in the K562 and MCF7 cells when compared to the A549 cells. Nevertheless, derivative 5b was less cytotoxic in comparison to derivatives 5, 5a and kulokekahelide-2. In short, SAR demonstrated that cyclic structure and the chirality of position 21 in Ala in kulokekahilide-2 were essential for its cytotoxicity, while the number of member was less important. Furthermore, addition of a halogen at the *para* position of the phenyl group in kulokekahilide-2 increased cytotoxic activity in A549, K562, and MCF7 cells [55].

**Figure 19:**
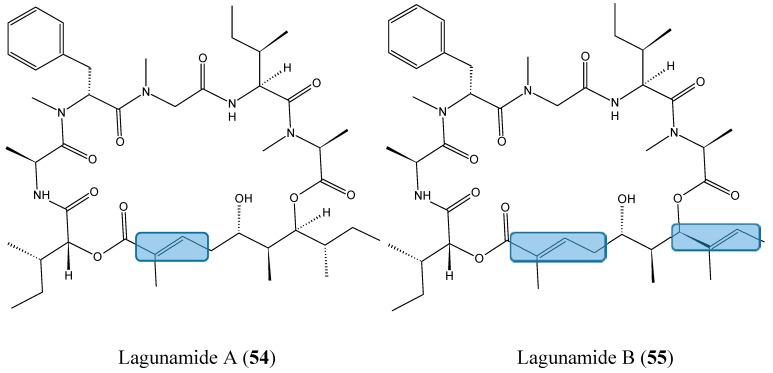
Lagunamide A and B.

Lagunamides A (**54**, Figure 19) and B (**55**, Figure 19), two cyclic depsipeptides structurally close to the aurilide-class of molecules, were recently isolated from the filamentous marine cyanobacterium, *Lyngbya majuscula*, obtained from the shallow lagoon at Pulau Hantu, Singapore [56]. Lagunamide A exhibited selective activity when tested against a panel of cell lines such as P388, A549, PC3, HCT8, and SK-OV3, with IC_50_ values ranging from 1.6 nM to 6.4 nM, whereas lagunamide B, with IC_50_ values of 20.5 and 5.2 nM, respectively in P388 and HCT8 cells, showed reduced cytotoxicity. Thus, it was assumed that the enhanced cytotoxicity of lagunamide A could be due to the presence of an olefinic group on the polyketide moiety. Biochemical studies using HCT8 and MCF7 cancer cells suggested that the cytotoxicity of the lagunamides might occur via induction of mitochondrial- mediated apoptosis [56].

#### 2.4.2. Cyclic heptapeptides

**Figure 20:**
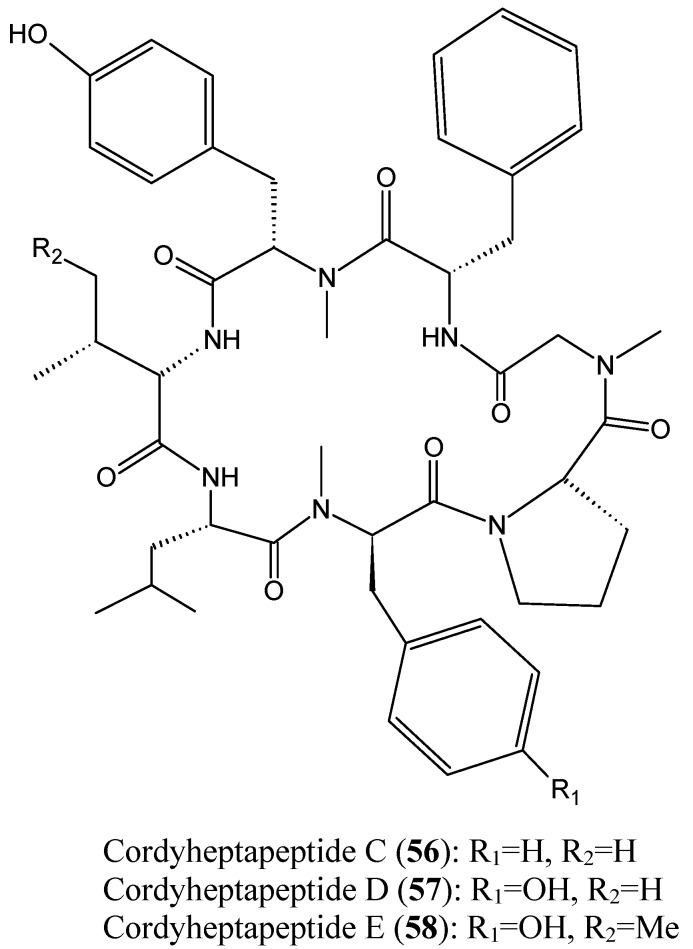
Cordyheptapeptide C, D and E.

Chen *et al.* [57] reported the isolation of three new cycloheptapeptides, cordyheptapeptides C-E (**56**–**58**, Figure 20), from the fermentation extract of the marine-derived fungus *Acremonium persicinum* SCSIO 115. The structural characteristics between the cordyheptapeptides contribute mostly to their biological activities. Thus, cordyheptapeptide E (**58**) exhibited potent cytotoxicity against human glioblastoma (SF-268), human breast cancer (MCF-7), and human lung cancer (NCI-H460) cell lines, with IC_50_ values of 3.2, 2.7, and 4.5 μM, respectively*.* Cordyheptapeptide **C** (**56**) displayed strong cytotoxicity against SF-268 and MCF-7 cells with IC_50_ values of 3.7 and 3.0 μM, respectively, and weaker cytotoxicity towards NCI-H460 cells (11.6 µM). Cordyheptapeptide D (**57**) was inactive against all the three cell lines tested [57].

#### 2.4.3. Sepia Ink Oligopeptide (SIO)

*Sepia* ink oligopeptide (SIO) is a tripeptide extracted first from *Sepia esculenta* by enzymolysis. SIO significantly inhibited the proliferation of DU-145, PC-3 and LNCaP prostate cancer cell lines in a time- and dose-dependent manner. SIO induces apoptosis in these cell lines, and the molecular mechanisms involved seemed to be related to the mitochondria-mediated pathway. In addition, SIO treatment induced strong S and G2/M phase cell cycle arrest in a dose-dependent manner in DU-145 and LNCaP cells, while it induced strong Sub G1 and G0/G1 phase cell cycle arrest in a dose-dependent manner in PC-3 cells [58].

#### 2.4.4. Hoiamide D

**Figure 21:**
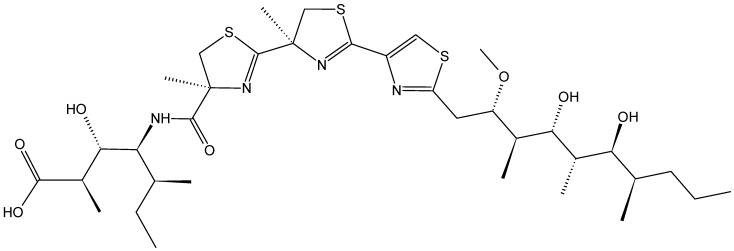
Structures of the carboxylate and acid forms of hoiamide D (**59**).

Hoiamide D (**59**, Figure 21) is a polyketide synthase-non-ribosomal peptide synthetase that was isolated in both its acid and carboxylate forms from two separate collections of the Papua New Guinea cyanobacterium *Symploca* sp. Hoiamide D exhibited potent inhibition of the p53/HDM2 protein binding, with an EC_50_ value of 4.5 µM and a minimal cytotoxicity to the mammalian H460 cell line at 40 µM, compared to control [59].

#### 2.4.5. Protuboxepin A

**Figure 22:**
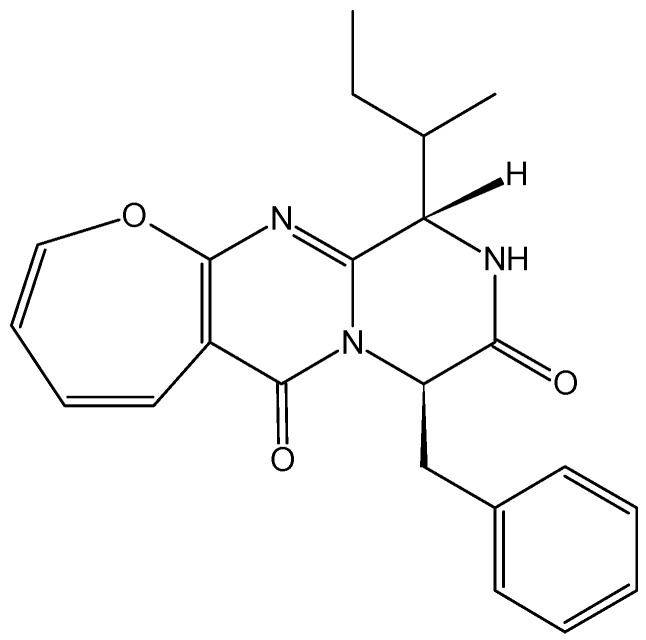
Protuboxepin A.

Protuboxepin A (**60**, Figure 22) is a new oxepin-containing diketopiperazine-type compound that was isolated from culture broth of the marine-derived fungus *Aspergillus* sp. SF-5044. Its anti-proliferative activity towards several cancer cell lines was previously described, and in 2012, Asami *et al.* [60] demonstrated that protuboxepin A induces cancer cell growth inhibition through a sequence of biochemical and morphological events. Protuboxepin A induces a rounded morphology, inhibits microtubule dynamics by preferentially microtubule-stabilizing, and specifically induces metaphase arrest and chromosome misalignment in tumor cells, which in turn induces apoptosis [60].

#### 2.4.6. Polypeptide CS5931

Cheng and colleagues [61] have extracted and purified a novel polypeptide, CS5931, with a molecular weight of 5931 Da from the ascidian *Ciona savignyi*. CS5931 exhibited strong cytotoxicity against several cancer cell lines, including human colon cancer (HCT-8 and HCT116), breast cancer (MCF-7), hepatoma (BEL-7402), cervical cancer (HeLa), and lung adenocarcinoma (A549) cell lines, in a time-dependent and dose-dependent manner. The polypeptide CS5931 significantly induced a G2/S phase arrest, with a decrease in G0/G1 phase population and treatment of HCT-8 cells with CS5931 disrupted the surface of the cell membrane, the mitochondrial transmembrane potential, and increased the amount of cytochrome C in the cytosol. Thus, CS5931 induces apoptosis via the activation of the mitochondrial pathway [61].

### 2.5. Phenols/Polyphenols

#### 2.5.1. Aeroplysinin-1

**Figure 23:**
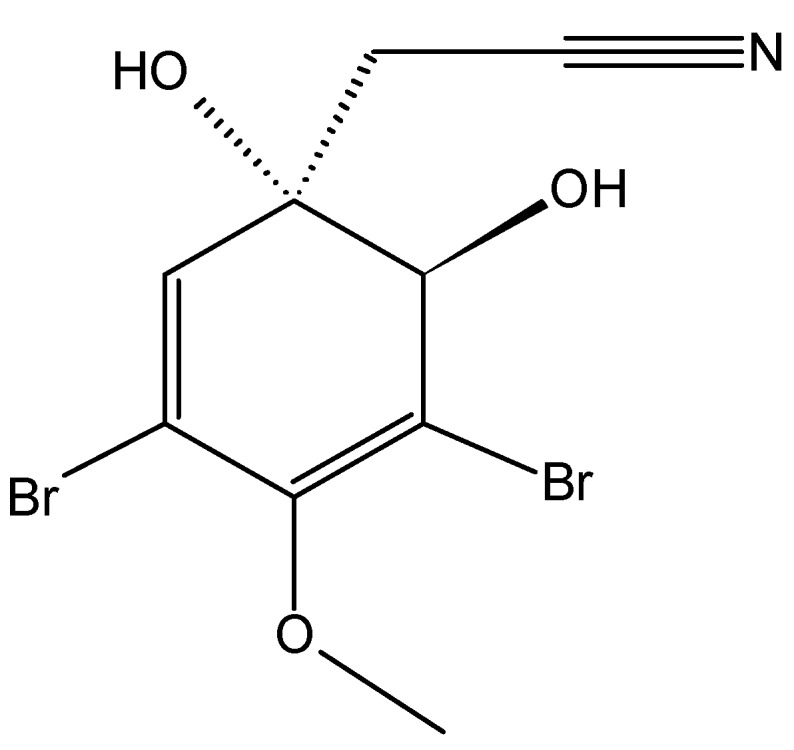
Aeroplysinin-1 (**61**).

Aeroplysinin-1 (**61**, Figure 23) is a naturally occurring brominated tyrosine metabolite that was extracted from the marine sponge *Aplysina aerophoba*. Aeroplysinin-1 inhibited, in a concentration-dependent manner, the growth of the endothelial cells (BAEC), colon carcinoma cells (HCT-116), and fibrosarcoma cells (HT-1080), with respective IC_50_ values of 2.1, 4.7, and 2.3 µM [62]. Treatment of endothelial cells with aeroplysinin-1 induces morphological and biochemical changes that include chromatin condensation and nuclear fragmentation, an increase in the percentage of cells with sub-diploid DNA content, the activation of caspases-2, -3, -8 and -9, and the cleavage of apoptotic substrates, such as PARP and lamin-A. Furthermore, the apoptosis-inducing mechanism of aeroplysinin-1 is endothelial cell-specific and dependent on the apoptogenic mitochondrial pathway through the activation of the BH3-only pro-apoptotic protein Bad and cytochrome*c* release [62].

#### 2.5.2. Bromophenol Bis (2,3-dibromo-4,5-dihydroxybenzyl) ether (BDDE)

**Figure 24:**
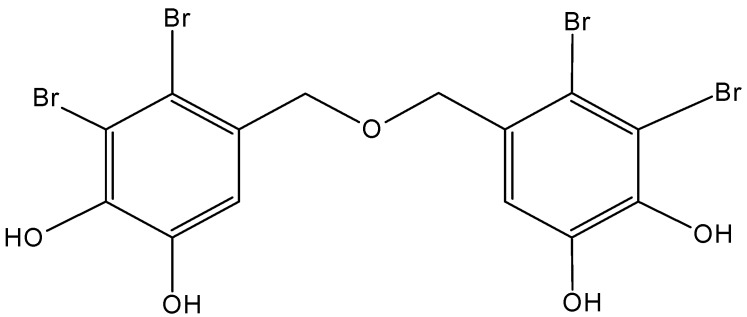
Bis (2,3-dibromo-4,5-dihydroxybenzyl) ether (BBDE, **62**).

Bis (2,3-dibromo-4,5-dihydroxybenzyl) ether (BDDE) is a marine bromophenol compound isolated from the marine algae *Leathesia nana* and *Rhodomela confervoides* [63]. BDDE (**62**, Figure 24) exhibited broad and potent cytotoxicity against several cancer cell lines (HeLa, HCT-116, HCT-8, SMMC-7721, A549, and K562 cells), and the most sensitive were the human myelogenous leukemia K562 cells, which were inhibited in a dose-dependent manner with an IC_50_ value of 13.9 µg/mL. BDDE induced S phase arrest and apoptosis in K562 cells via the mitochondrial pathway. Moreover, BDDE binds to the DNA minor groove and inhibits topoisomerase I through interaction with its catalytic cycle [63].

#### 2.5.3. Diphlorethohydroxycarmalol

**Figure 25:**
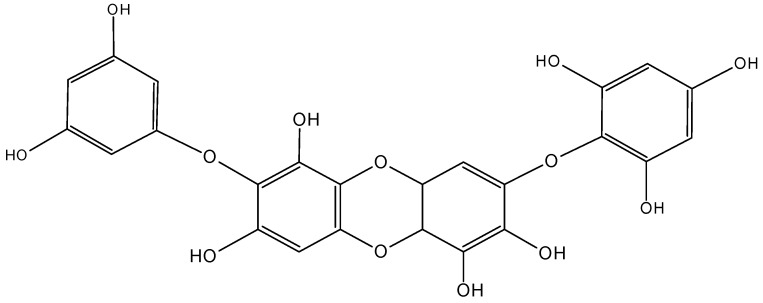
Diphlorethohydroxycarmalol (DC, **63**).

Diphlorethohydroxycarmalol (DC, **63**, Figure 25) is a type of phlorotannin polyphenol compound that was isolated from *Ishige okamurae*, an edible brown alga collected along the coast of Jeju Island, Korea [64,65]. Kang and associates [64] demonstrated that DC strongly inhibited the growth of the promyelocytic leukemia cell line HL60. This inhibitory effect resulted in the induction of apoptosis via mitochondrial dysfunction and cell cycle arrest. This was evidenced by the reduction of mitochondrial membrane potential, accumulation of sub-G_1_ cell population, and the regulation of the expression of caspase-3, PARP, and Bcl-2 family proteins.

#### 2.5.4. C-glycoside Angucyclines

**Figure 26:**
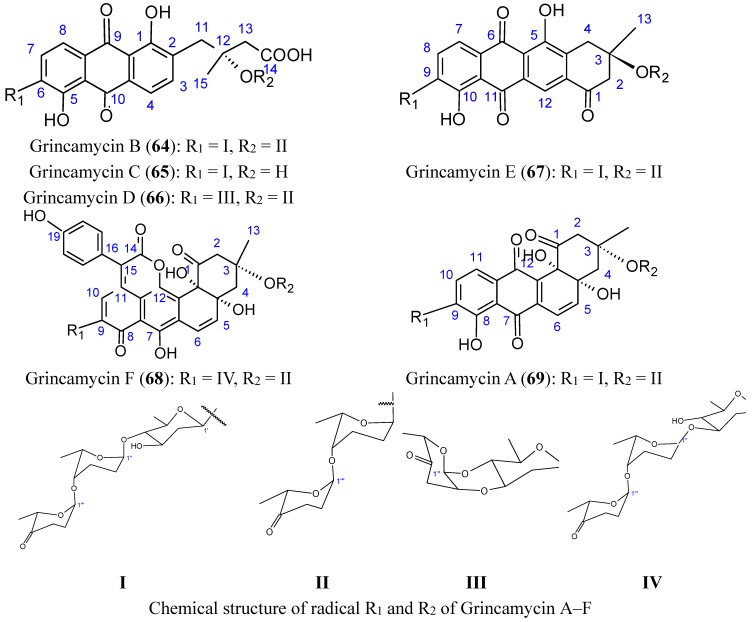
Grincamycin A, B, C, D, E, F.

Huang *et al.* [66] isolated five new C-glycoside angucyclines, named grincamycins B, C, D, E, F (**64**–**68**, Figure 26), and the previously reported grincamycin A (**69**, Figure 26), a known angucycline antibiotic from *Streptomyces lusitanus* SCSIO LR32, an actinomycete of deep-sea origin.

The grincamycins B-E (compounds **64**–**67**) have exhibited *in vitro* cytotoxicity against the human hepatoma cell line HepG2, human pancreatic cell line SW-1990, human cervical cancer cell line HeLa, human lung cancer cell line NCI-H460, human breast cancer cell line MCF-7, and the mouse melanoma cell line B16, with IC_50_ values ranging from 1.1 to 31 μM. Grincamycin A (**69**) was the most potent, and grincamycin F (**68**) was only cytotoxic towards the human breast cancer cell line MCF-7 (IC_50_ = 19 µM). It was suggested that the enlarged aglycone of grincamycin **68** containing a six-membered lactone ring and a hydroxybenzene, in addition to the typical angucycline, cancels cytotoxicity [66].

### 2.6. Polysaccharides

**Figure 27:**
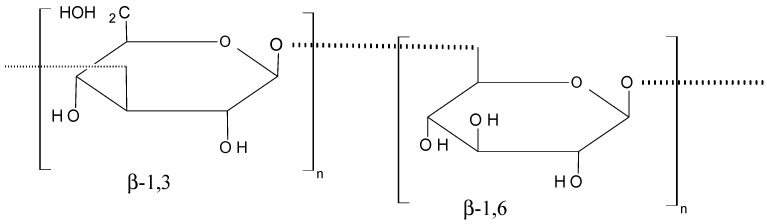
Laminarin (**70**).

Laminarin (**70**, Figure 27) is a marine glucan that was obtained from *Laminaria japonica* Aresch (Laminariaceae) and *Ecklonica kurome* Okam. (Alariaceae), which consists of a main linear chain of 15 to 35 glucopyranose units joined by β-(1→3) with β (1→6)-linkages [67,68]. In their report, Park *et al.* [67] demonstrated that laminarin inhibits cell growth in a dose-dependent manner and induces apoptosis in the HT-29 human colon cells. The proposed mechanism involves cell death pathways and the insulin-like growth factor-I receptor (IGF-IR), whose overexpression plays a part in cancer cell proliferation and survival. Ji *et al.* [68] reported that laminarin also induces apoptosis in human colon cancer (LoVo) cells via a mitochondrial pathway.

### 2.7. Quinones

#### 2.7.1. Prenylated Bromohydroquinones

**Figure 28:**
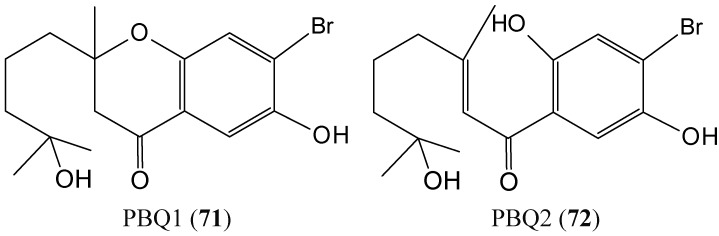
7-Hydroxycymopochromanone (PBQ1, **71**) and 7-hydroxycymopolone (PBQ2, **72**).

Two prenylated bromohydroquinones compounds, 7-hydroxycymopochromanone (PBQ1) and 7-hydroxycymopolone (PBQ2) (**71**–**72**, Figure 28) were isolated from the marine algae *Cymopolia barbata*, which was collected from the shoreline of the north eastern coast of Jamaica [69]. PBQ2 selectively impacted the viability of the colon cancer cell line HT29, with an IC_50_ value of 19.82 ± 0.46 μM, whereas PBQ1, its structural isomer, had no significant impact on any of the cell lines investigated. The presence of a tertiary hydroxyl group on PBQ2 appears to be essential for its bioactivity. PBQs 1 and 2 potently inhibited the activity of CYP1A1 with IC_50_ values of 0.39 ± 0.05 and 0.93 ± 0.26 μM, respectively. PBQ2 exhibited potency against the activity of CYP1B1 (IC_50_ = 0.14 ± 0.04 μM), which is known to be a universal marker for cancer and a target for drug discovery. These results highlight the PBQs as attractive candidates with potential for development as chemopreventive agents [69].

#### 2.7.2. SZ-685C

**Figure 29:**
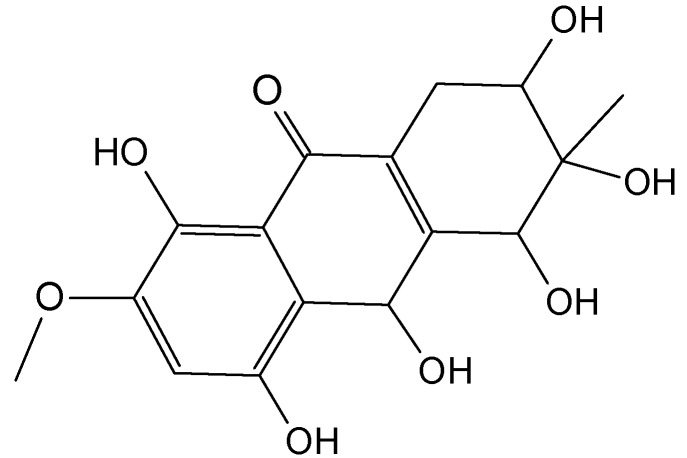
SZ-685C (**73**).

SZ-685C (**73**, Figure 29), with structural similarity to anthracycline, the most commonly used anti-cancer drug in the clinic, is a natural and a biologically active substance that was isolated from the secondary metabolites of the mangrove endophytic fungus No. 1403, collected from the South China Sea [70,71]. SZ-685C potently inhibited the proliferation of three cancer cells lines derived from breast cancer (MCF-7), human erythromyeloid leukemia (K562), and human promyelocytic leukemia (HL-60), with IC_50_ values of 7.38, 1.09 and 1.94 µM, respectively. Further, SZ-685C inhibited the proliferation of the adriamycin (ADR) resistant cell lines of these human cancer cells (IC_50_ = 4.17, 1.35 and 1.76 µM, respectively for MCF-7/ADR, K562/ADR and HL-60/ADR cells). Thus, SZ-685C decreased the ADR resistance factor from 19.19 to 0.57 in the MCF-7/ADR cells. Furthermore, SZ-685C exhibited a marked, dose-dependent inhibition of the survival of MCF-7/Akt cells (IC_50_ = 3.36 µM), in which the constitutively phosphorylated Akt conferred MCF-7 cell resistance to ADR. SZ-685C induced apoptosis in ADR-resistant breast cancer cells through both the intrinsic and extrinsic pathways. The systemically delivery of the marine anthraquinone SZ-685C was able to inhibit the growth of ADR-resistant tumors xenografts in nude mice with no detectable toxic effects, such as signs of discomfort or weight loss. As the activation of Akt by HER2/PI3K confers chemoresistance in breast cancer, these data support the potential usefulness of combining SZ-685C with other therapeutics in combating MDR in breast cancer chemotherapy [71].

#### 2.7.3. Hydroanthraquinones and Anthraquinones Dimers

**Figure 30:**
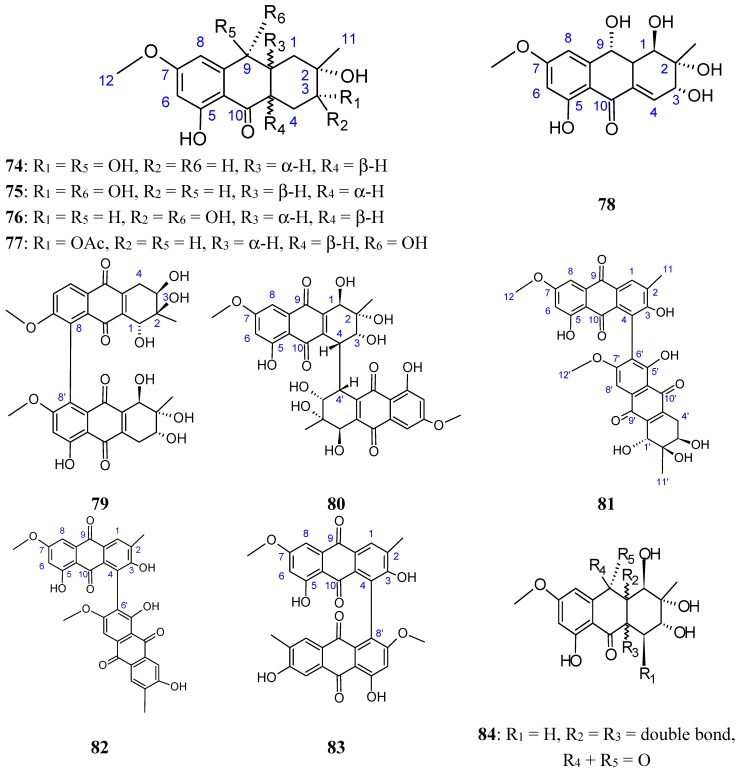
Hydroanthraquinones and anthraquinones dimers (**74**–**84**).

Zheng *et al.* [72] have isolated five new hydroanthraquinone derivatives, tetrahydroaltersolanols **74**−**77** (Figure 30) and dihydroaltersolanol A (**78**, Figure 30), five new alterporriol-type anthranoid dimers, alterporriols N-R (**79**−**83**, Figure 30), together with seven known analogues, tetrahydroaltersolanol B, altersolanol B, altersolanol C (**84**, Figure 30), altersolanol L, ampelanol, macrosporin, and alterporriol C from the culture broth and the mycelia of *Alternaria* sp. ZJ-2008003, a fungus obtained from a *Sarcophyton* sp. soft coral collected from the South China Sea. Interestingly, of the alterporriols compounds, only compound **80** contains the first isolated alterporriol dimer with a C-4−C-4' linkage.

Among the monomeric anthranoids, altersolanol C (**84**), which possesses aparaquinone moiety, exhibited potent cytotoxic activity towards the human colon carcinoma HCT-116, human breast cancer MCF-7/ADR, human prostatic cancer PC-3, and human hepatoma HepG2 and Hep3B cell lines with IC_50_ values ranged from 2.2 to 8.9 μM, whereas the hydroanthraquinone derivatives with an oxidized C-10 and a reduced C-9 fragment, were inactive (for instance compounds **74**–**78**). Moreover, among the alterporriol-type dimers, only alterporriol **81** was found to possess cytotoxic activity against PC-3 and HCT-116 cells, with IC_50_ values of 6.4 and 8.6 μM, respectively [72].

#### 2.7.4. Alterporriol L

**Figure 31:**
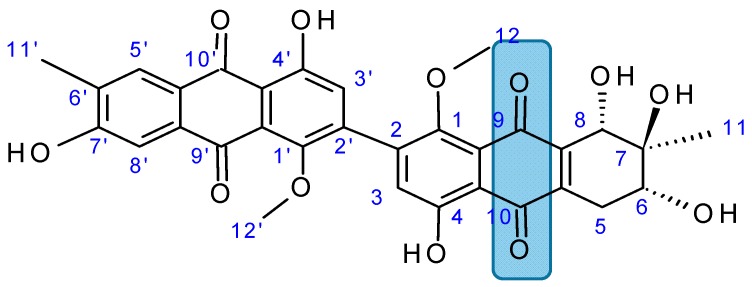
Alterporriol L (**85**).

Alterporriol L (**85**, Figure 31), a new marine bianthraquinone derivative, was isolated from a mangrove endophytic fungus, *Alternaria* sp. ZJ9-6B [73]. Alterporriol L demonstrated an attractive coupling position at C-2-C-2', and its substructure was closely related to epiadriamycin, an anticancer drug used widely in the clinic. Alterporriol L exhibited a dose-dependent inhibition of growth in the human breast cancer cell lines MCF-7 and MDA-MB-435, with IC_50_ values of 20.04 and 13.11 μM respectively. The morphological alterations and biochemical events induced by alterporriol L involve changes in the cytoskeleton and nuclear morphology, a significant increase of ROS and intracellular calcium, and loss of the mitochondrial membrane potential (MMP) in the MCF-7 cells. Alterporriol L induced apoptosis and necrosis in a dose-dependent manner in MCF-7 cells [73].

#### 2.7.5. Xestoquinones

**Figure 32:**
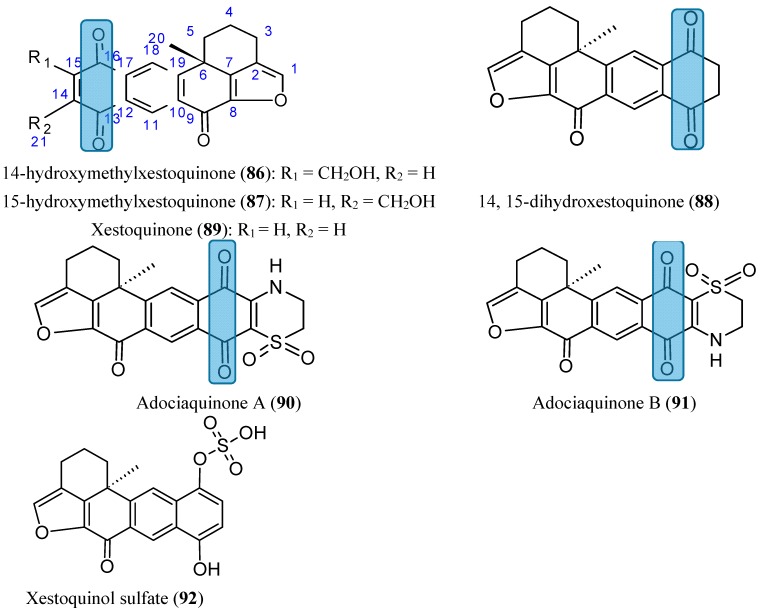
Xestoquinones.

Seven xestoquinones (**86**–**92**, Figure 32), including three new compounds, 14-hydroxymethyl-xestoquinone (**86**), 15-hydroxymethylxestoquinone (**87**), and 14, 15-dihydroxestoquinone (**88**), were isolated from a lipid extract of the marine sponge *Petrosia alfiani* [74]. Compounds **86**−**91** exhibited more potent cytostatic/cytotoxic effects in the human breast tumor T47D cells (IC_50_ values ranged from 2.6 to 15.9 µM) than in the human breast tumor MDA-MB-231 (IC_50_ values ranged from 6.1 to 28.1 µM). Moreover, these compounds demonstrated distinct abilities to inhibit HIF-1, with improved inhibition in the T47D cells. The hydroxymethylated metabolites **86** and **87** inhibited both hypoxia-induced and chemical hypoxia-induced HIF-1 activation with comparable low micromolar IC_50_ values (1.2–2.3 μM), whereas xestoquinone **89** and its 14, 15-saturated analogue **88** exhibited reduced potency (IC_50_ values 4.2–12.3 μM). The adociaquinones (**90** and **91**) potently and selectively suppressed iron chelator-induced HIF-1 activation with IC_50_ values of 0.2 and 0.2 μM, respectively. The 3,4-dihydro-2H-1,4-thiazine-1,1-dioxide moiety present in these compounds appears to be important for this enhanced selectivity toward chemical hypoxia-induced HIF-1 activation. Xestoquinol sulfate (**92**), a more polar compound, did not suppress cell proliferation and/or viability in the cell lines used, and had no effect on the HIF-1 activation. Thus, the 1,4-quinone moiety in compounds **86**−**91** seems to be an essential pharmacophoreto suppress HIF-1 activation [74].

#### 2.7.6. Aminoquinones

**Figure 33:**
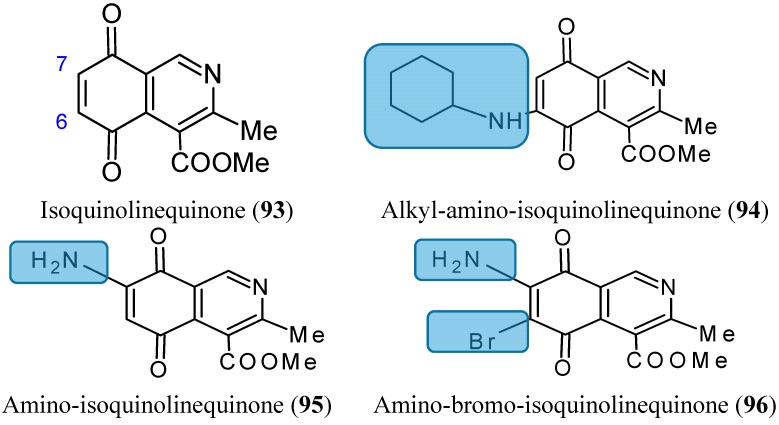
Aminoquinones and derivatives **93**–**96**.

The amino-, alkylamino- and alkylamino-haloisoquinolinequinone derivatives, structurally related to marine isoquinolinequinones, were obtained from isoquinolinequinone (**93**, Figure 33) by means of synthetic transformations These novel aminoquinones have demonstrated moderate to significant potency against MRC-5 (healthy lung fibroblasts), AGS (gastric adenocarcinoma), SK-MES-1 (lung cancer), J82 (bladder carcinoma), and HL-60 leukemia cells. Compounds **94**, **95** and **96** (Figure 33) were the most significant antitumor members, and compound **96** was selected as a promising lead compound due to its high potency on the tested tumor cell lines (IC_50_ values in the 0.21 µM–0.49 µM range) and high selectivity index as compared to the anti-cancer agent etoposide. According to the SAR studies, it appears that insertion and substitution of the nitrogen and halogen atoms at the quinone nucleus of the isoquinolinequinone pharmacophore improves the antitumor potency compared with the precursor (**93**, Figure 33). The activity was more important for those members containing the cyclohexylamino group at C-6 (compound **94**), the amino group at C-7 (compound **95**) and the amino and bromine substituents at the 6- and 7-positions (compound **96**) [75].

#### 2.7.7. Bostrycin and Deoxybostrycin analogs

**Figure 34:**
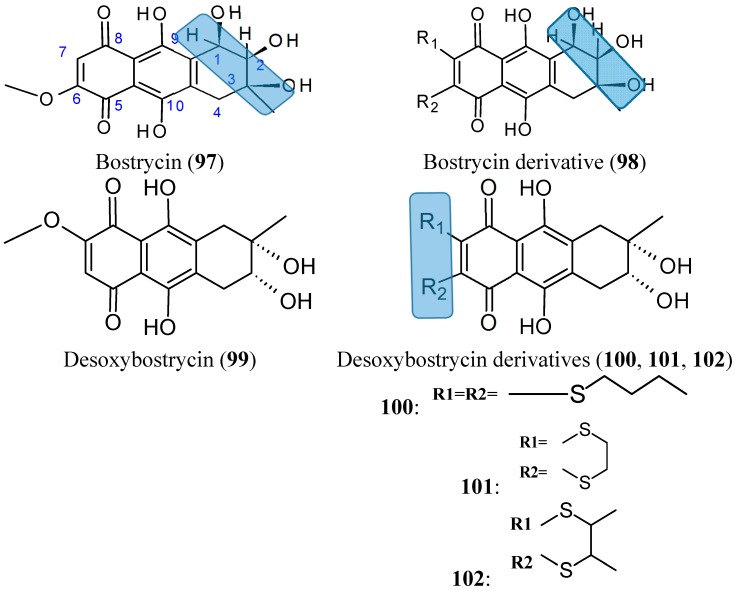
Bostrycin, deoxybostrycin and derivatives **97**–**102**.

Bostrycin (**97**, Figure 34) is a natural tetrahydroanthraquinone compound that was isolated from the mangrove endophytic fungus No. 1403 collected from the South China Sea. In 2012, Chen and colleagues [76] synthesized and evaluated the cytotoxicity of a series of new derivatives from bostrycin. Among these derivatives, compound **98** (Figure 33) showed a broad-spectrum of antitumor activity, with equal potency to epirubicin against the following cancer cell lines: human breast MCF-7 and MDA-MB-435, human lung A549, human liver HepG2, and human colon HCT-116, with IC_50_ values of 0.57, 0.63, 0.37, 0.82, and 0.68 μM, respectively. These values were 7.0-fold higher than bostrycin (with IC_50_ values of 2.18, 2.82, 2.63, 7.71, and 4.78 μM, respectively). Although, some modified compounds displayed potent cytotoxicity over the parent compound bostrycin, the majority of compounds also exhibited marked cytotoxicity against the human breast epithelial cell line, MCF-10A. The SAR analysis indicated that the enhanced cytotoxicity was achieved by dioxylcarbonyl groups at C-2 and C-3 positions, tertiary amino groups at C-6 position and dithiol-substituted derivatives at C-6 and C-7 positions of bostrycin [76].

From deoxybostrycin (**99**, Figure 34) an anthraquinoneisolated from the mangrove endophytic fungus *Nigrospora* sp. No. 1403, Chen *et al.* [77] synthesized 21 new derivatives by modifying deoxybostrycin at the C-2, C-3, C-6, and C-7 positions. The *in vitro* cytotoxicity of all the new compounds evaluated against MDA-MB-435, HepG2, and HCT-116 cancer cell lines indicated that most of these derivatives possessed significant cytotoxic activity, with IC_50_ values ranging from 0.62 to 10 μM. Among all the derivatives, compound **101** (Figure 33), which was characterized by a relatively rigid 2, -dihydro-1,4-dithiine heterocycle attached to deoxybostrycin, had the highest potency against all the three tested cancer cell lines and displayed a comparable cytotoxic activity with the positive control epirubicin against MDA-MB-435 cells, with an IC_50_ value of 0.62 μM *versus* 0.56 μM for epirubicin. SAR studies showed that the cytotoxicity was substantially improved with the introduction of alkylthio groups at the C-6 and C-7 positions of the deoxybostrycin. In particular, compounds **100**, **101**, and **102** (Figure 34) displayed the highest cellular cytotoxicity against MDA-MB-435 and might serve as valuable source of new potent anti-tumor agents.

### 2.8. Sterols and Steroids

#### 2.8.1. 9,11-Secosterol

**Figure 35:**
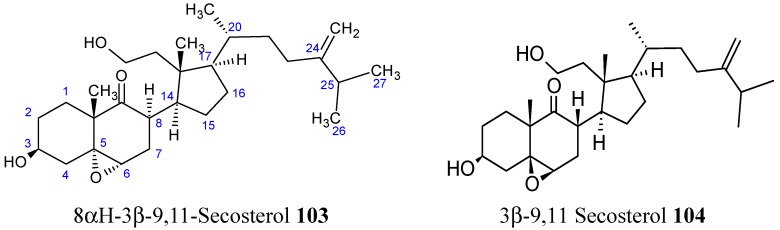
Secosterol analogs **103**, **104**.

Chemical investigations of the EtOAc-soluble fraction from the EtOH extract of Formosa soft coral *Sinularia granosa* afforded the new 9,11-secosteroid compound **103** (Figure 35), along with a known metabolite **104** (Figure 35).

Compound **103** exhibited potent cytotoxicity against human cervical epithelial carcinoma (HeLa), human laryngeal carcinoma (HEP2), human medulloblastoma (Daoy), and human breast adenocarcinoma (MCF-7), with ED_50_ values of 8.21, 6.21, 5.53 and 4.99 µg/mL, respectively, whereas compound **104** was found to be cytotoxic only against Daoy and MCF-7 cancer cell lines, with ED_50_ values of 7.07 and 9.98 µg/mL [78].

#### 2.8.2. Polyoxygenated Steroids

**Figure 36:**
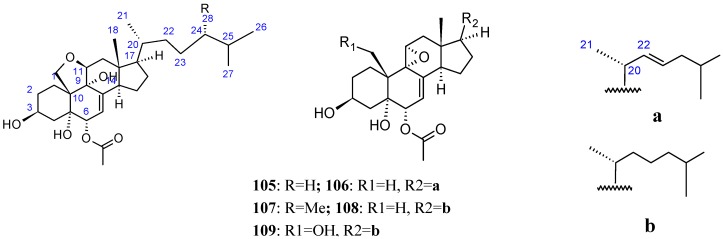
Polyoxygenated steroid dysideasterol analogs.

The bioassay-guided fractionation of an active organic extract obtained from an Okinawan marine sponge of the genus *Dysidea* afforded three new polyoxygenated steroids dysideasterols (**105**–**107**, Figure 36), along with two known related compounds (**108**–**109**, Figure 36). Compounds **104**–**109** demonstrated stronger cytotoxicity against human epidermoid carcinoma A431 cells, with respective IC_50_ values of 0.23, 0.3, 0.2, 0.15, and 0.3 µM. Although a characteristic structural feature in compounds **106**, **108** and **109** was an allylic epoxide that underwent ring opening to give a tetrahydrofuran ring in **105** and **107**, the biological result suggested that the allylic epoxide moiety was not responsible for the cytotoxic activity [79].

#### 2.8.3. 11-Dehydrosinulariolide

**Figure 37:**
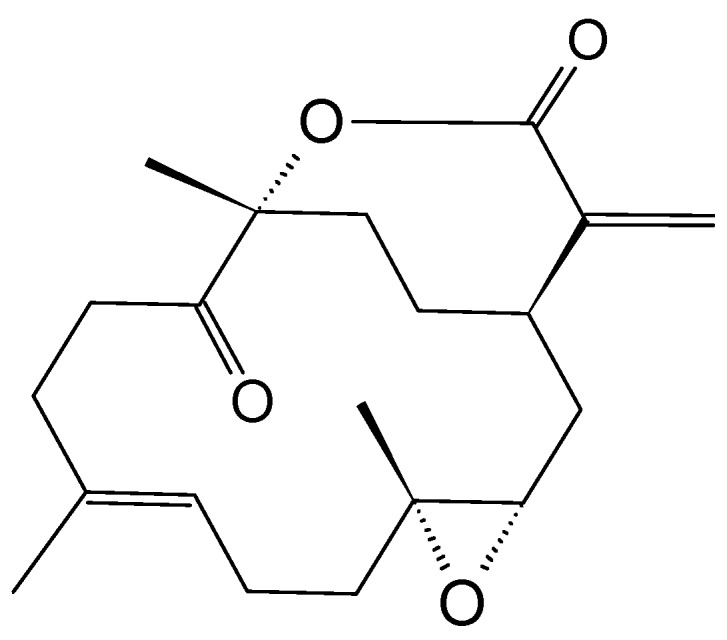
11-Dehydrosinulariolide (**110**).

11-Dehydrosinulariolide (**110**, Figure 37), an active compound isolated from the soft coral *Sinularia leptoclados*, was reported to possess anti-tumor effects in the oral squamous cell carcinoma cell line CAL-27 [80]. The MTT assay, flow cytometry, and migration assay demonstrated that 11-dehydrosinulariolide was strongly cytotoxic to the oral squamous cell carcinoma cells. 11-dehydrosinulariolide mainly exerts its anti-tumor effects through the induction of early and late apoptosis pathways. Moreover, the fact that proteomic studies have identified some proteins involved in the mitochondrial dysfunction and ER-stress pathway suggests that 11-dehydrosinulariolide induces cell apoptosis through either mitochondrial dysfunction-related or ER stress pathway. The results highlight the 11-dehydrosinulariolide to be a potential anticancer drug for oral cancer treatment [80,81].

#### 2.8.4. Diketosteroid (*E*)-Stigmasta-24(28)-en-3,6-dione

**Figure 38:**
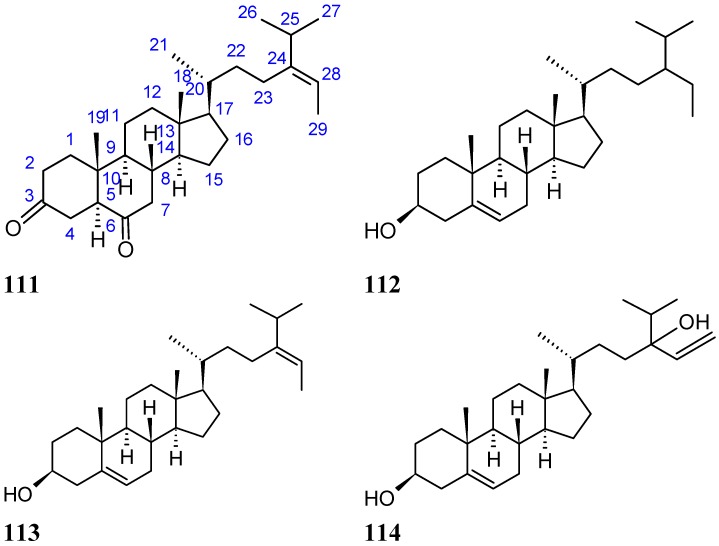
Diketosteroid, (*E*)-stigmasta-24(28)-en-3,6-dione (**111**), β-sitosterol (**112**), fucosterol (**113**) and saringosterol (**114**).

A new diketosteroid, (*E*)-stigmasta-24(28)-en-3,6-dione (**111**, Figure 38), together with three known steroids, β-sitosterol (**112**, Figure 38), fucosterol (**113**, Figure 38), and saringosterol (**114**, Figure 38), was isolated from marine green alga *Tydemania expeditionis* collected in the China Sea. Compound **111** exhibited moderate inhibitory activities against the prostate cancer cell lines DU145, PC3, and LNCaP, with IC_50_ values of 31.27, 40.59, and 19.80 μM, respectively, while compound **113** was the more active compound, with IC_50_ values of 12.38, 2.14, and 1.38 μM, respectively. Compound **114** was inactive on DU145 and PC3 cells, and exhibited only weak inhibitory activityin LNCaP cells. The SAR between these steroids showed that the hydroxyl group at C-3 (compound **113**) increased the cytotoxic activity, whereas the hydroxyl group at C-24 (compound **113**) decreased activity. Furthermore, compound **111** exhibited significant affinity for the androgen receptor, with an IC_50_ value of 7.19 μM, whereas compounds **113** and **114** were inactive. Thus, there might be other mechanisms of action involved in the cytotoxic activity [82].

### 2.9. Terpenes and Terpenoids

#### 2.9.1. 10-Acetylirciformonin B

**Figure 39:**
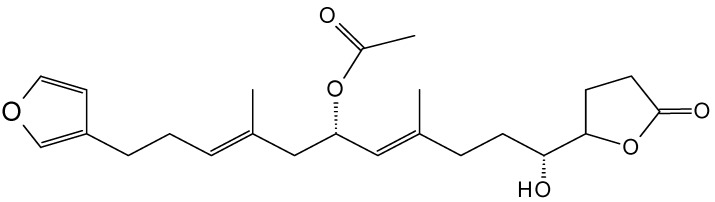
10-Acetylirciformonin B (**115**).

10-Acetylirciformonin B (**115**, Figure 39) is a furanoterpenoid that was isolated and purified from marine sponge *Ircinia* sp. This linear C22-sesterterpenoid potently inhibited the growth of leukemia HL60 cells, with an IC_50_ value of 1.7 µg/mL obtained at 48 h of treatment. The anticancer activity of 10-acetylirciformonin B was exerted through the induction of DNA damage and apoptosis. Induction of DNA damage was mediated by the phosphorylation of histone H2A.X and p-CHK2 (checkpoint kinase), sensitive markers of DNA double-strand breaks (DSBs), and apoptosis was triggered through activation of caspases-8, -9, and -3, leading to PARP cleavage and the down-regulation of Bcl-xL and the up-regulation of Bax [83].

#### 2.9.2. Phenazine Analogs

**Figure 40:**
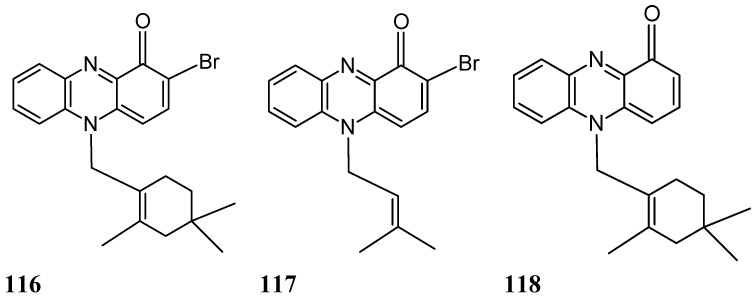
Phenazineanalogs **116**–**118**.

Two new brominated terpenoid phenazines, N-substituted brominated monoterpene phenazine (**116**, Figure 40) and N-substituted isoprenylated phenazine (**117**, Figure 40), along with lavanducyanin (**118**, Figure 40), a known phenazine derivative, were isolated and identified from the fermentation broth of a marine-derived *Streptomyces* sp. (strain CNS284). Compounds **116**, **117**, and **118** dose-dependently inhibited TNF-α-induced NF-κB activity, with IC_50_ values of 4.1, 24.2, and 16.3 μM, respectively and reduced lipopolysaccharide (LPS)-induced prostaglandin E2 (PGE2) production with IC_50_ values 7.15, 0.89, 0.63 μM, respectively. Phenazines **117** and **118** were more active in inhibiting nitric oxide (NO) production, with IC_50_ values of 15.1 and 8.0 μM, respectively. Moreover, treatment of cultured HL-60 cells with various concentrations of the phenazines led to dose-dependent accumulation in the sub-G1 compartment after a 24 h incubation period, indicative of apoptosis [84]. Phenazine **118** demonstrated the greatest potency in inducing apoptosis, followed by phenazines **116** and **117**. Otherwise, phenazines **116** and **117** potently inhibited human cyclooxygenase 2 (COX2) activity, with IC_50_ values of 4.0 and 7.2 µM, respectively. The N-substituent pattern, as well as bromination at C-2 (phenazine **116**), enhanced NF-κB inhibitory activity, although this effect was not correlated with a greater reduction of NO or PGE2 production. As with the NF-κB and inducible nitric oxide synthase (iNOS) pathways, an abnormal elevation of COX-2 and PGE2 are likely to play an important role in promoting carcinogenesis through cellular proliferation, suppression of apoptosis, and enhancement of angiogenesis and invasiveness. Thus, their inhibition might be of value for the treatment of human ailments, such as cancer [84].

#### 2.9.3. Diterpenoid Compounds

##### Echinohalimane A

**Figure 41:**
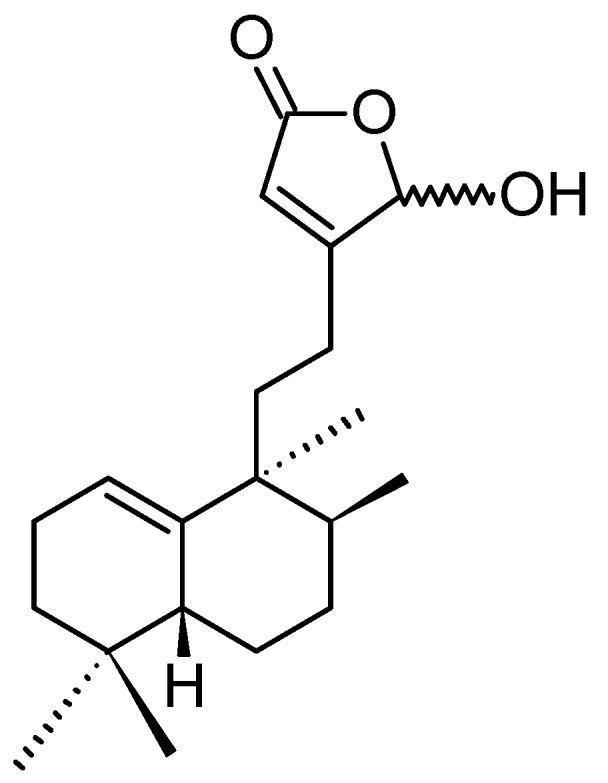
Echinohalimane A (**119**).

Echinohalimane A (**119**, Figure 41) is the first halimane analogue from marine organisms belonging to the phylum Cnidaria. This diterpenoid was isolated from a Formosan gorgonian *Echinomuricea* sp. Echinohalimane A exhibited potent cytotoxicity towards LoVo (human colorectal adenocarcinoma), DLD-1 (human colorectal adenocarcinoma), MOLT-4 (human acute lymphoblastic leukemia), and HL-60 (human acute promyelocytic leukemia) cells, with respective IC_50_ values of 0.563, 0.967, 2.111, 2.117 µg/mL [85].

##### (1*S*,2*S*,3*E*,7*E*,11*E*)-3,7,11,15-Cembratetraen-17,2-olide

**Figure 42:**
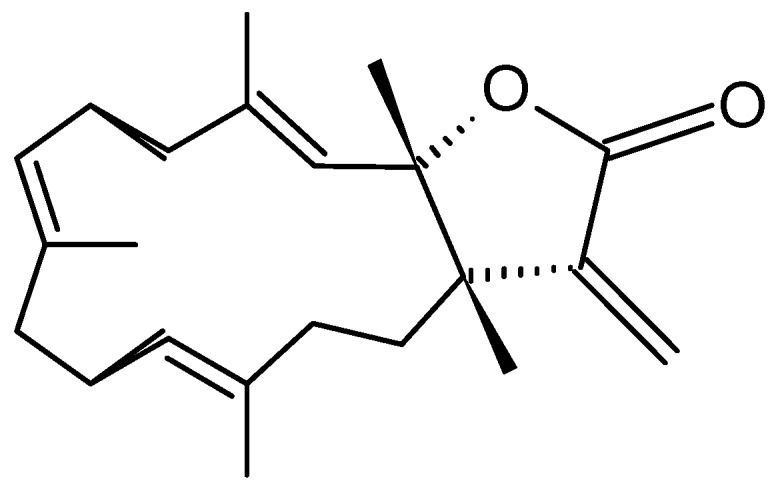
(1*S*,2*S*,3*E*,7*E*,11*E*)-3,7,11,15-Cembratetraen-17,2-olide (**120**).

LS-1 or (1*S*,2*S*,3*E*,7*E*,11*E*)-3,7,11,15-cembratetraen-17,2-olide (**120**, Figure 42), was isolated from the EtOAc layer of the Vietnamese marine soft coral *Lobophytum* species by bioassay-guided fractionation [86]. LS-1 significantly inhibited the proliferation of HT-29 human colon carcinoma cells in a dose-dependent manner with an IC_50_ value of 3.7 μM. Further, treatment with 5 and 10 μM LS-1 significantly reduced colony formation. LS-1 exhibited its anticancer activity through the induction of apoptosis via ROS generation in human colon cancer cells. This was evidenced by disruption of mitochondrial membrane potential, cytosolic release of cytochrome c, sub-G1 peak accumulation, increases in apoptotic bodies, activation of Bid, caspase-3, -8, and -9, and cleavage of PARP, along with the suppression of B cell lymphoma-2 (Bcl-2) expression. ROS production induced by LS-1 contributes to the down-regulation of p-Akt, p-Src, and p-STAT3, increased the phosphorylation of JNK, and decreased the phosphorylation of p38 and ERK. Moreover, LS-1 inhibits the expression of catalase and glutathione peroxidase, thereby leading to an accumulation of ROS and cell death. These results provide support for the development of LS-1 as a potent therapeutic agent against human colon cancer [86].

##### Michaolides

**Figure 43:**
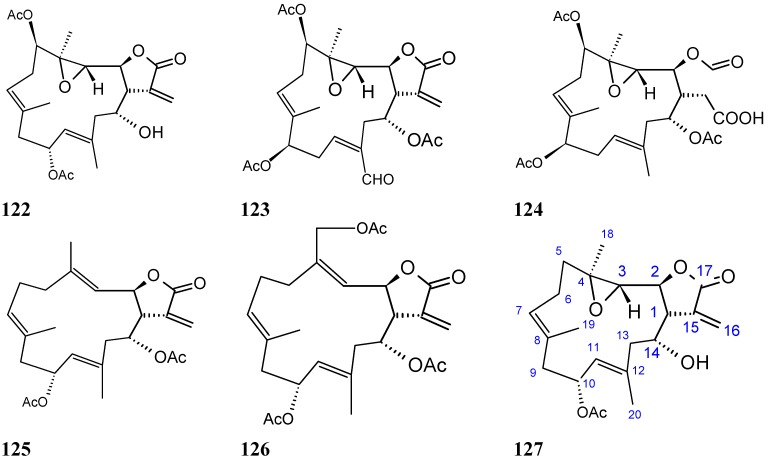
Michaolides and derivatives **121**–**127**.

Wang and Duh [87] reported the isolation of six new cembranolides, michaolides L–Q (**121**–**126**, Figure 43), together with a known cembranolide, lobomichaolide (**127**, Figure 43) from the CH_2_Cl_2_ extract of the soft coral *Lobophytum michaelae*. These compounds exhibited significant cytotoxicity against HT-29 (human colon adenocarcinoma), P-388 (mouse lymphocytic leukemia), A-549 (human lung epithelial carcinoma) tumor cells, and human embryonic lung (HEL) cells, with ED_50_ values ranging from 0.3 to 4.9 µM for all the compounds except for **124**. Thus, it was suggested that the α-exo-methylene-γ-lactone moiety plays an important role in the cytotoxicity [87].

#### 2.9.4. Triterpenoid Compounds

##### Stellettin A

**Figure 44:**
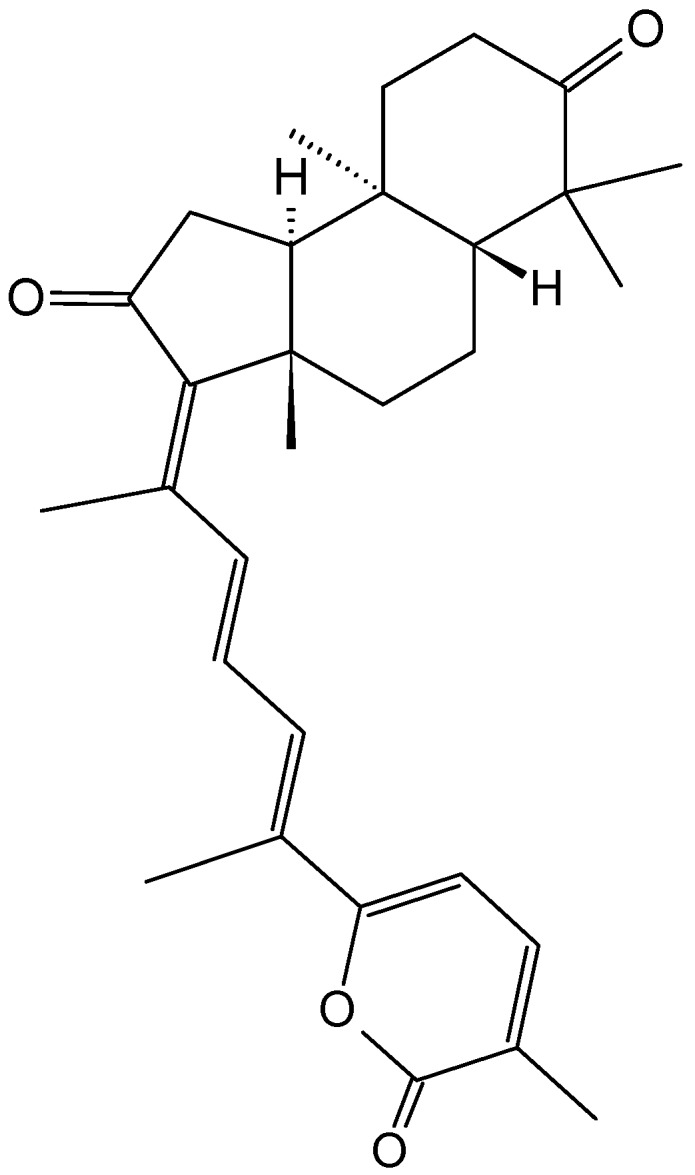
Stellettin A (**128**).

Stellettin A (**128**, Figure 44) is an isomalabaricane triterpenoid that was obtained from a South China Sea sponge, *Geodia japonica*. After 48 h of treatment, stellettin A significantly inhibited the growth of the murine B16F10 melanoma cells (IC_50_ = 0.15 μg/mL) and the murine Leydig TM3 cells (IC_50_ = 0.8 μg/mL), whereas the human immortalized HaCaT keratinocytes, human colon HT29 carcinoma cells, and murine melan-a melanocytes were much less sensitive [88]. The underlying mechanism may involve the induction of ER stress and accumulation of unfolded proteins, such as tyrosinase (TYR) and tyrosinase-related protein 1 (TRP-1), two melanoma marker proteins. Finally, cells undergo autophagy as it was evidenced by the conversion of an ubiquitin-like molecule, LC3I (microtubule-associated protein 1 light chain 3) to LC3II, a protein that is localized specifically in the autophagic structures [88].

##### Triterpene glycosides

**Figure 45:**
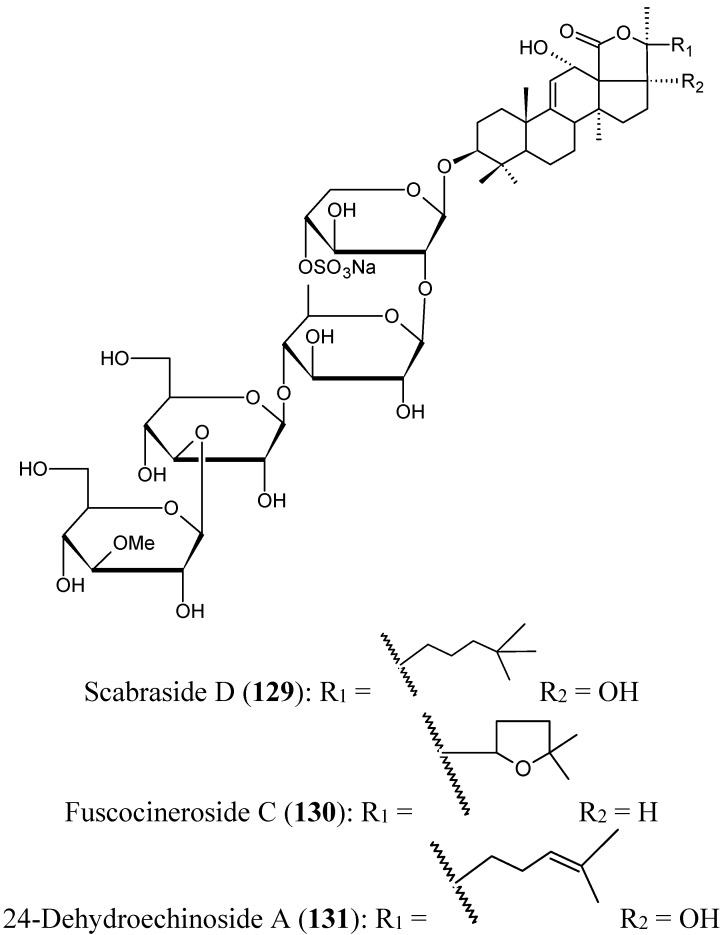
Triterpene glycosides **129**–**131**.

Han’s group have isolated from the cucumber *Holothuria scabra*, collected from offshore water of Hainan Island in the South China Sea, three triterpene glycosides identified as scabraside D, a new sulfated triterpenoid glycoside (**129**, Figure 45), fuscocineroside C (**130**, Figure 45), and 24-dehydroechinoside A (**131**, Figure 45). All these compounds exhibited strong cytotoxic activity against mouse leukemia cells (P-388), human lung cancer cells (A-549), gastric cancer cells (MKN-28), human colorectal cancer cells (HCT-116), and human breast cancer cells (MCF-7) with IC_50_ values ranging from 0.93 to 2.60 μmol/L [89].

#### 2.9.5. Sesquiterpene Compounds

##### Hirsutanol A

**Figure 46:**
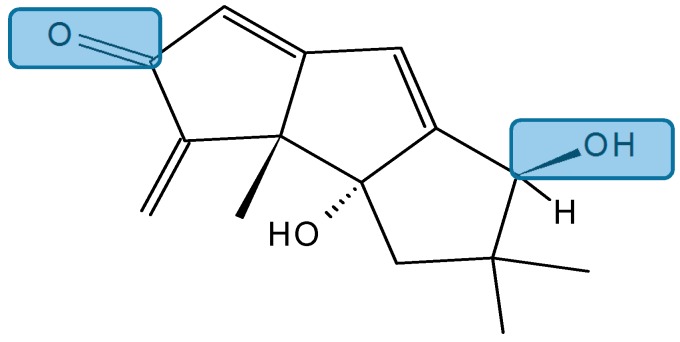
Hirsutanol A (**132**).

Hirsutanol A (**132**, Figure 46), a sesquiterpene compound isolated from the marine fungus *Chondrostereum* sp. in the coral *Sarcophyton tortuosum*, significantly inhibited the human breast cancer cell line MCF-7 with an IC_50_ value of 10.69 µM, obtained after treatment for 72 h. The anticancer molecular mechanisms of hirsutanol A involve the increase of intrinsic ROS, mainly hydrogen peroxide H_2_O_2_, induction of apoptosis, and autophagy via accumulation of ROS in MCF-7 cells. Moreover, blocking autophagy with a specific inhibitor, bafilomycin A1 or Atg7-siRNA, remarkably enhanced the cell growth inhibition and apoptosis induced by hirsutanol A. Thus, treatment with hirsutanol A in combination with inhibitors of autophagy may exhibit a synergistic effect. These results showed that hirsutanol A may be a promising lead compound and deserves further investigation as a potential anticancer agent [90].

##### Chondrosterins

**Figure 47:**
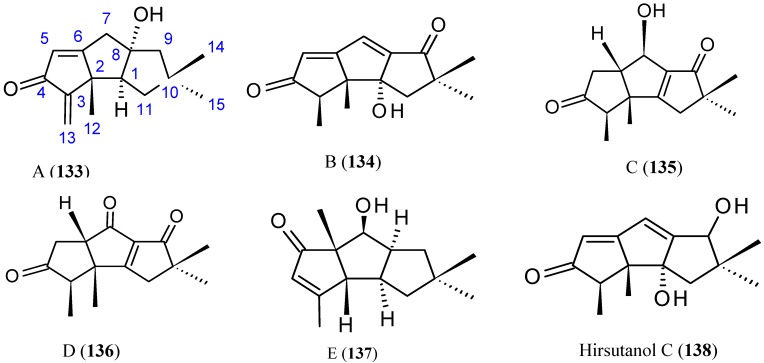
Chondrosterins A–E and hirsutanol C (**133**–**138**).

Li *et al.* [91] have isolated five new sesquiterpenoids, chondrosterins A–E (**133**–**137**, Figure 47), and the known compound hirsutanol C (**138**, Figure 47), from the marine fungus *Chondrostereum* sp., collected from the inner tissue of soft coral *Sarcophyton tortuosum* in the South China Sea. Chondrosterins **133**–**136** and **138** are hirsutane-type sesquiterpenoids, while compound **137** has a novel rearranged hirsutane skeleton, which could be derived by migration of a methyl group from C-2 to C-6. Chondrosterin A (**133**), with the typical α-methylene ketone group, demonstrated significant cytotoxic activity against the human lung cancer cell line A549, human nasopharyngeal carcinoma cell line CNE2, and human colon cancer cell line LoVo, with IC_50_ values of 2.45, 4.95, and 5.47 μM, respectively, whereas compounds **134**–**138** were inactive (IC_50_ > 200 μM) [91].

**Figure legends**: Each chemical structure (compounds **1**–**138**) was drawn using the software ChemBioDraw Ultra, version 12.0.3.1216 (Cambridge Soft Corporation, Cambridge, MA, USA) and is in conformity with the original structures published in the literature. In some cases, if the pharmacophores are known, they are highlighted in the form of blue color.

## 3. Discussion

**Figure 48:**
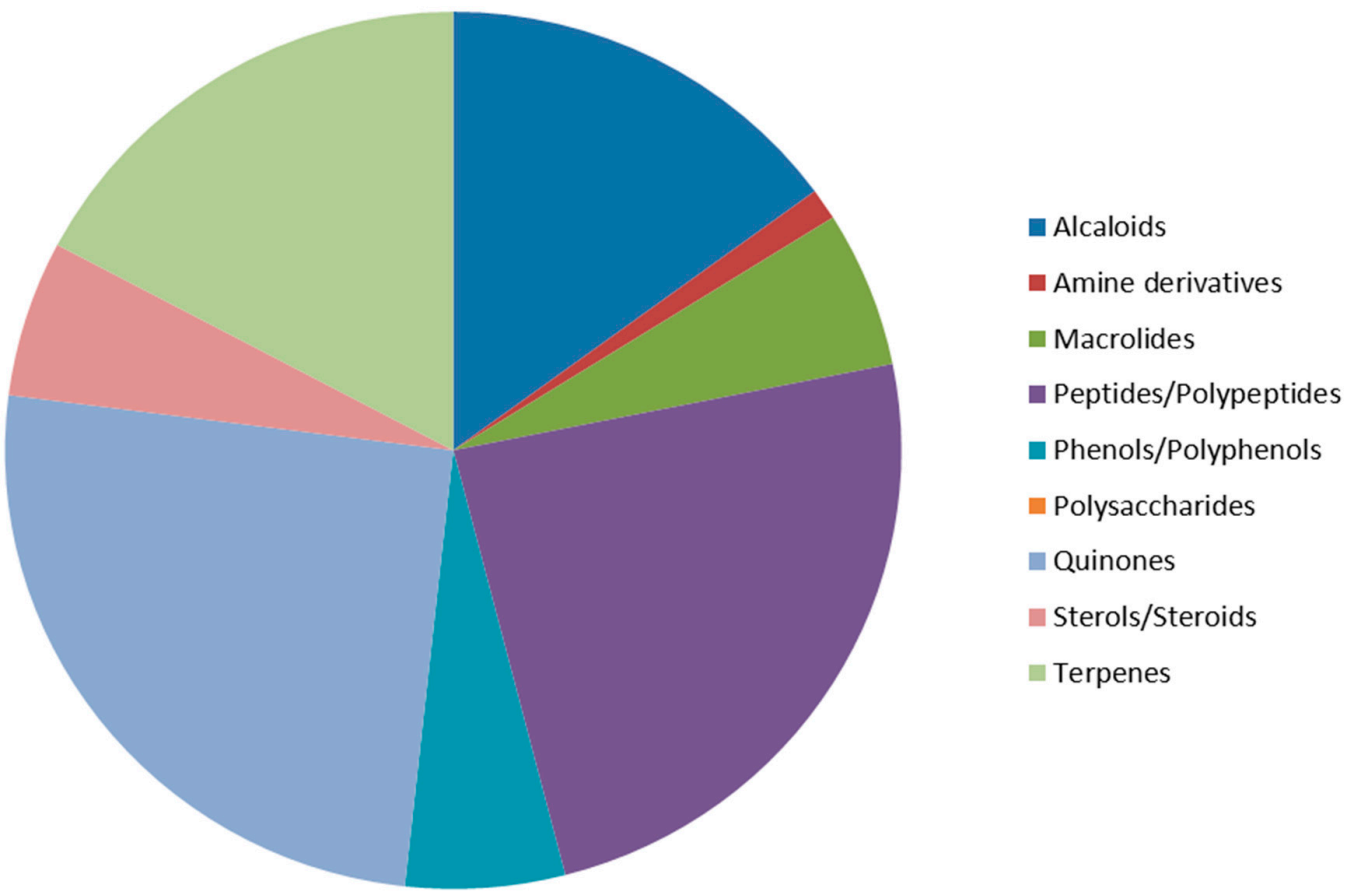
Anticancer compounds regrouped per family.

According to our survey, 87 molecules (62%) published in 2012 seem to be promising anticancer drugs, along with 53 known compounds. These compounds are regrouped into nine classes of chemicals, including alkaloids, amines, macrolides, peptides/polypeptides, phenols/polyphenols, polysaccharides, quinones, steroids, and terpenes. Among these new compounds, quinones were the most represented (25.3%), followed by peptides/polypeptides (24.1%), terpenes (17.2%), and alkaloids (14.9%) (Figure 48).

The isolated compounds account for about 69%, and were from various marine organisms, including actinomycetes, ascidians, cyanobacteria, mollusks, sponges, and tunicates. On the other hand, 31% were obtained by synthetic transformations.

Among the new compounds, some remarkable examples of marine bioactive compounds seem to be promising anticancer compounds. Thus, the aminoquinone **96** and halichoblelide C showed high selective cytotoxic activity with respect to other anticancer agents. The neamphamides and the derivatives 5 and 5a of kulokekahilide-2, with a significant anti-cancer activity at nanomolar low concentrations against a wide range of cancer cells also appear to be other promising cytotoxic compounds. Because of ethical, medical, and economic limitations and constraints on the number of patients eligible for clinical trials, most of the research has to be done in experimental systems [92]. Moreover, according to our survey, the biological mechanism of action remains unknown for 79.3% of compounds tested *in vitro.* Further*,*to the best of our knowledge, none were proposed for clinical trial and *in vivo* study. Of course, the discovery of new cancer therapeutics holds great promise for many patients, but it is worth noting the fact that, despite successful in preclinical study, several novel drugs fail in early clinical trials [93]. Furthermore, many compounds that showed interesting activities *in vitro* lose these properties *in vivo* because of interactions between the compound and the tested organism, including adsorption, distribution, metabolism and excretion [94]. The biological mechanisms involved in the anticancer properties of the investigated compounds involve the inhibition of various targets such as tubulin, multidrug resistant protein (P-gp), proteasome, kinases, NF-κB. Some compounds exert their cytotoxicity activity through the induction of endoplasmic reticulum stress and inhibition of COX-2 and iNOS expression. Finally, cells undergo apoptosis through caspase activation, MMP depolarization, Bcl-xL, Bax and PARP cleavage, cytochrome c release, increasesin the intracellular concentration of Ca^2+^ and intrinsic ROS, Bcl-2, and Akt down-regulation. Other compounds, such as stellettin A and hirsutanol A, can trigger autophagy. Besides these biological mechanisms, novel mechanisms of action were reported for eribulin, which inhibits microtubule dynamics through a mechanism different from other known compounds such as the taxanes. In addition, a novel mechanism of resistance to peloruside A and laulimalide was identified, and its involvement in the down-regulation of vimentin, which has various cellular properties such as cell signaling, cell division, cell survival, apoptosis, migration and in the regulation of intermediate filament structure and dynamics cell.

## 4. Conclusions

The data presented in this review indicate the great value of natural marine products, as well as their synthetic analogs. The physico-chemical conditions of the oceans are such that most marine organisms contain a variety of bioactive compounds with unique structural features. This review demonstrated the potential for marine natural products as promising sources of anticancer compounds. The compounds described here exhibited their anticancer properties through different mechanisms, such as cell cycle arrest, anti-inflammatory activity, apoptosis, and/or autophagy. Thus, the isolation or modification of novel marine products, as well as their analogs, and the subsequent evaluation of their bioactivity through standardized experimental methods will allow for the development of promising new chemotherapeutic drugs. With this, we can expect more antitumor compounds of marine origin can finally be used as new clinical agents with a high positive risk-benefit ratio and low side effects.

## Abbreviations

### Cell lines

A549: Lung carcinoma cell line
AGS: Human stomach adenocarcinoma cell line
B16: Mouse melanoma cell line
BEL-7402: Human hepatoma cancer cell line
CEM: leukemia cell
CNE2: Human nasopharyngeal carcinoma cell line
Daoy: Human medulloblastoma cells
DLCL: Diffuse large cell line
DU-145: Prostate cancer cell line
H1299: Lung cancer cells
HCC: Human hepatocellular carcinoma cell line
HCT116: Human colon carcinoma cell line
HCT116: Human colon carcinoma cell line
HCT-8: Human colon cancer cell line
HEL: Human embryonic lung
HEL: Human embryonic lung cell line
HeLa: Human cervical epithelial carcinoma
HEp 2: Human laryngeal carcinoma cells
HepG2: Human hepatocellular liver carcinoma cell line
HL-60: Human promyelocytic leukemia cell line
HT-29: Human colon adenocarcinoma cell line
HT29: Human colon carcinoma cells
IGROV1: Human ovarian carcinoma cells
IGROV1-DXR: Doxorubicin-resistant cell line
J82: Bladder carcinoma cells
K562: Human chronic myeloid leukemia cell line
KB: Human nasopharynx carcinoma cell line
LNCaP: Prostate cancer cell line
LoVo: Colon cancer cell line
MCF-10A: Human breast epithelial cell line
MCF-7: Human breast adenocarcinoma cell line
MDA-MB231: Mammary carcinoma cell line
MDA-MB-231: Mammary carcinoma cell line
MiaPaCa2: Pancreatic cancer cells
MKN-28: Gastric cancer cells
MRC-5: Healthy lung fibroblasts
NCI-H460: Human lung cancer cell line
P-388: Murine lymphocytic leukemia cell line
PBMC: Peripheral blood mononuclear cells
PC-3: Prostate cancer cells
S180: Murine sarcoma cell line
SF-268: Human glioblastoma cells
SK-MES-1: Lung cancer cell line
SNU-423: Hepatocellular carcinoma cell line
SW-1990: Human pancreatic cell line
UCI-101: Ovarian cancer cells

### Other

ABC transporters: ATP-binding cassette transporters
ADR: Adriamycin
Bcl-2: B-cell lymphoma 2
Bax: Bcl-2-associated X protein
BDDE: Bis (2, 3-dibromo-4, 5-dihydroxybenzyl) ether; BF65 and BF78: Hemiasterlin derivatives
cDDP: Cisplatin
C-L: Caspase-like
CLL: Chronic lymphocytic leukemia
COX-2: Cyclooxygenase 2
CT-L: Chymotrypsin-like
DC: Diphlorethohydroxycarmalol
DMBPO: 5-(2, 4-dimethylbenzyl) pyrrolidin-2-one
EGFR: Epidermal Growth Factor Receptor
ER: Endoplasmic reticulum
ERK: Extracellular signal-regulated kinase
HDAC: Histone deacetylase
IGF-IR: Insulin-like growth factor-I receptor
JNK: c-Jun N-terminal kinase
MDR: Multi-Drug Resistance
MED: Mycoepoxydiene
MMP: Mitochondrial membrane potential
MTD: Maximum tolerated dose
MTT: 3-(4,5-dimethylthiazol-2-yl)-2,5-diphenyl-2*H*-tetrazolium bromide
NF-κB: Nuclear factor-kappa B
NO: Nitric oxide
PARP: Poly (ADP-ribose) polymerase
PGE2: Prostaglandin E2
P-gp: P-glycoprotein
PKC: Protein kinase C
**PLA** and **Lau**: Peloruside A and Laulimalide
PP2A: Protein phosphatase 2A
ROS: Reactive oxygen species
SAR: Structure-activity relationships
SERCA: Sarcoplasmic-ER Ca^2+^-ATPase
T-L: Trypsin-like
TNF-α: Tumor necrosis factor
TRP-1: Tyrosinase-related protein 1
TYR: Tyrosinase
VEGFR 2: Vascular Endothelial Growth Factor Receptor 2
